# Supplementary material for: Homo-PROTACs: bivalent small-molecule dimerizers of the VHL E3 ubiquitin ligase to induce self-degradation
Source: Nat Commun. 2017 Oct 10;8:830. doi: 10.1038/s41467-017-00954-1 (PMC5635026; doi:10.1038/s41467-017-00954-1)
Supplement: Supplementary file 1 — Supplementary Information [file 41467_2017_954_MOESM1_ESM.pdf]

Supplementary Figures

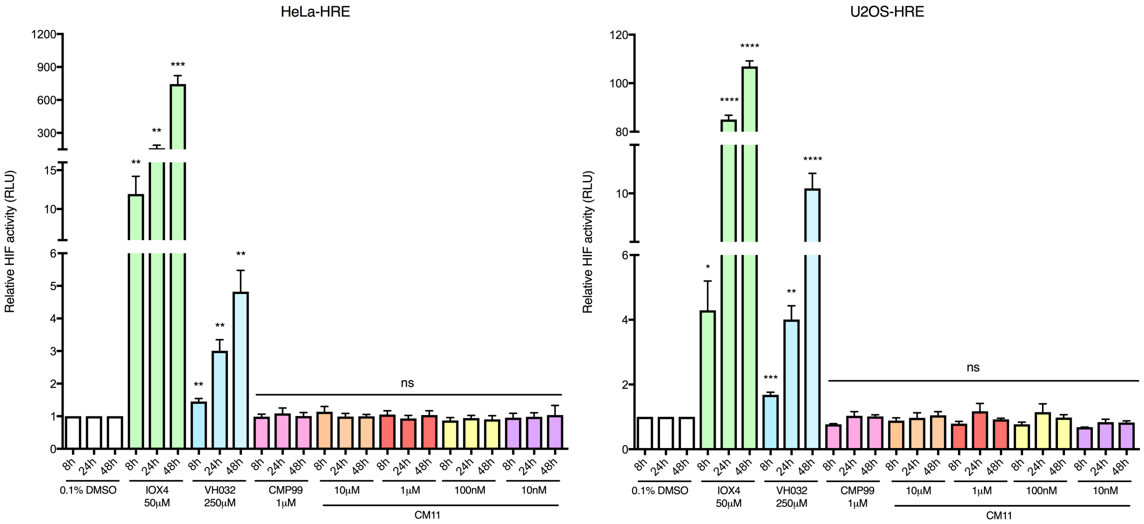

Supplementary Figure 1: HeLa or U2OS cells stably expressing HRE-luciferase reporter plasmid were treated with the indicated compounds at the indicated concentrations for the indicated time. The data shown represent the mean ± s.e.m. (n=3 independent biological replicates). Statistical significance was determined with one-tailed t tests: *P < 0.05; **P < 0.01; ***P < 0.001; ****P < 0.0001; n.s. not significant.

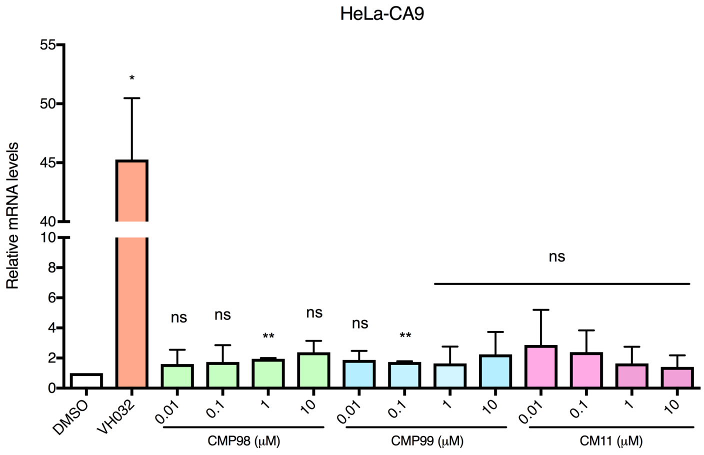

Supplementary Figure 2: Dose-response curve of CA9 mRNA expression in HeLa (16h). The data shown represent the mean ± s.e.m. (n=3 independent biological replicates). Statistical significance was determined with one-tailed t tests: *P < 0.05; **P < 0.01; ***P < 0.001; n.s. not significant.

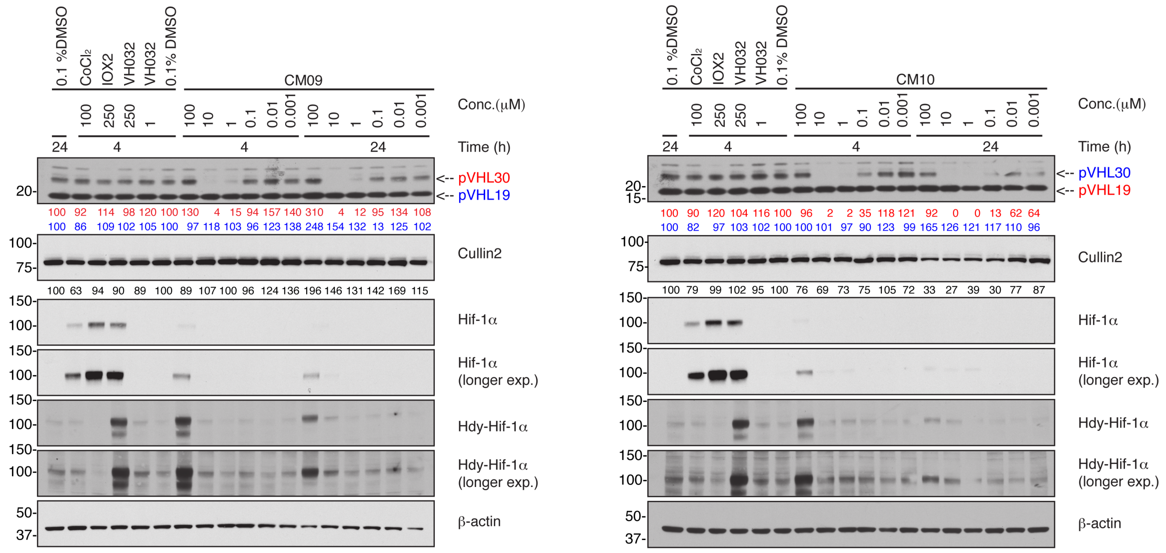

Supplementary Figure 3: Hela cells were treated with increasing concentration of indicated compound for 4 h or 24 h.

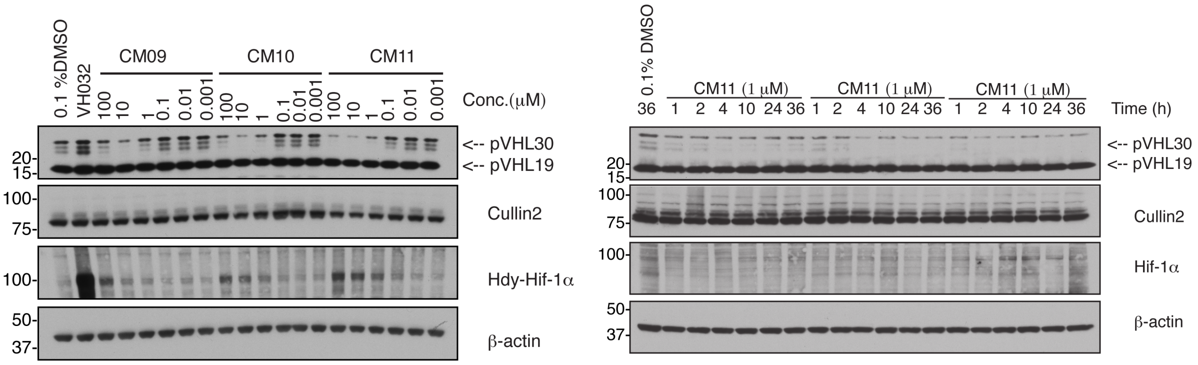

Supplementary Figure 4: Concentration dependency experiment in U2OS (10 h treatment)(left) and Time course experiments of lysate from U2OS (right).

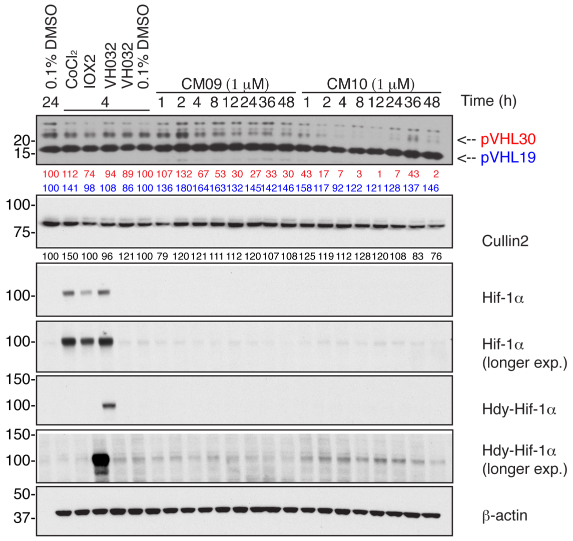

Supplementary Figure 5: Time-course immunoblots of lysates from HeLa cells subjected to 0.1% DMSO, $CoCl_2$ (100 μM), IOX2 (150μM), VH032 (250 μM or 1 μM) or 1 μM of indicated compounds.

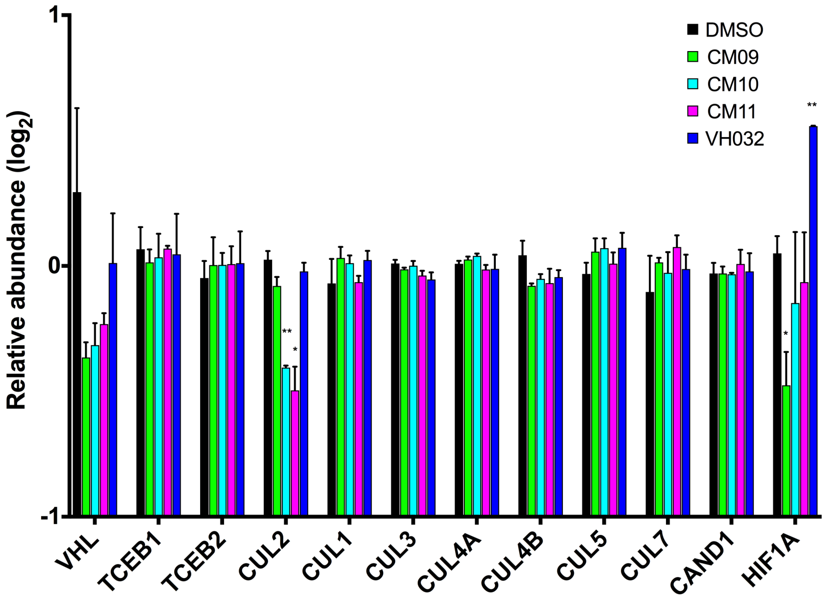

Supplementary Figure 6: Quantified levels of proteins from TMT-labeling proteomics experiments are plotted as fold change (log2) for the indicated compound treatment. Data shown are mean (±1 s.e.m.) from two replicates. Statistical significance of relative protein abundance compared to DMSO was assessed by two-tailed t-test; $*P < 0.05$, $**P < 0.01$, $***P < 0.001$. Note: VHL quantification was based on two unique peptides (SLVKPENYR 168-176; and VVLPVWLNFDGEPQPYPTLPPGTGR 83-107, see Supplementary Data 1). These peptides are present in all VHL isoforms.

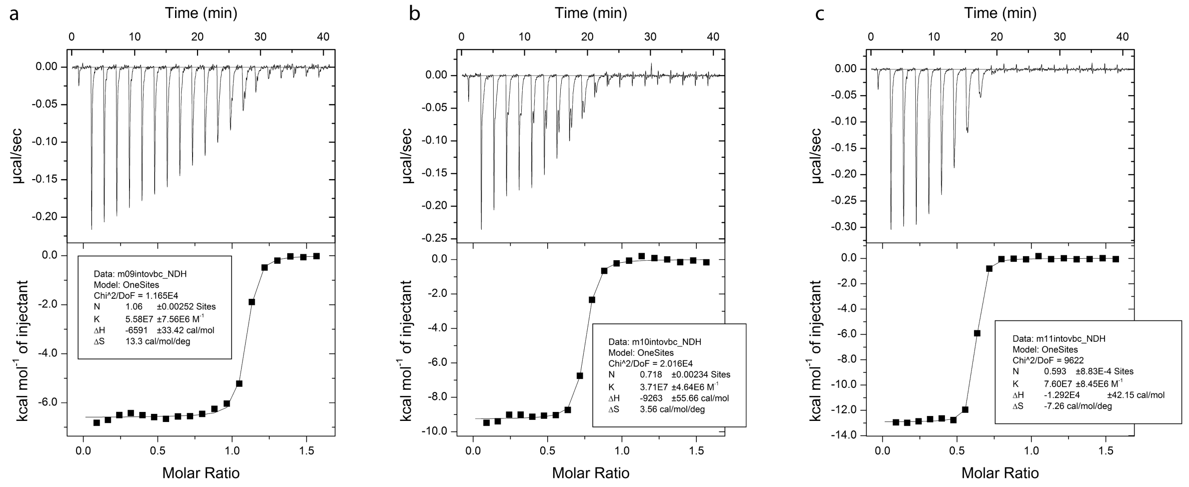

Supplementary Figure 7: Integrated ITC heat curves of CM09 (a), CM10 (b), and CM11 (c) against $V_{19}CB$.

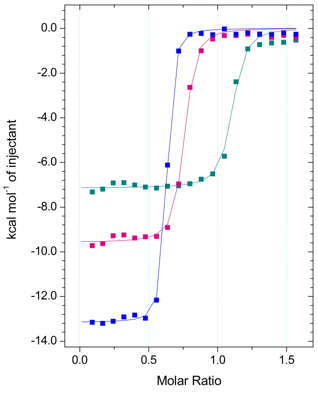

Supplementary Figure 8: Superposition of the integrated ITC heat curves of CM11 (blue), CM09 (cyan) or CM10 (magenta) titrations against V_{19}CB.

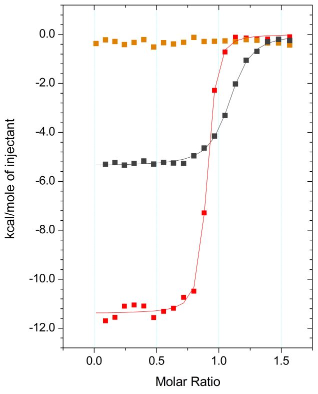

Supplementary Figure 9: Superposition of the integrated ITC heat curves of CM11 (red), CMP98 (orange) or CMP99 (grey) titrations against V_{30}CB.

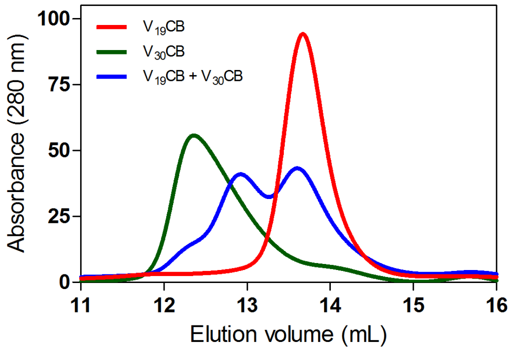

Supplementary Figure 10: SEC assay of complex formation after incubation of CM11 with V_{19}CB(red), CV_{30}CB (green) or both (blue) .

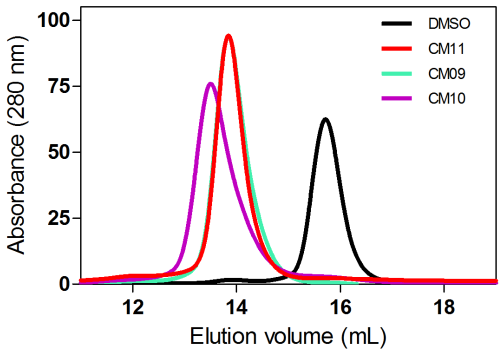

Supplementary Figure 11: SEC assay of complex formation after incubation of CM11 (red), CM09 (green), CM10 (blue) or DMSO (black) with $V_{19}CB$.

Supplementary Figure 12

Fig. 4a

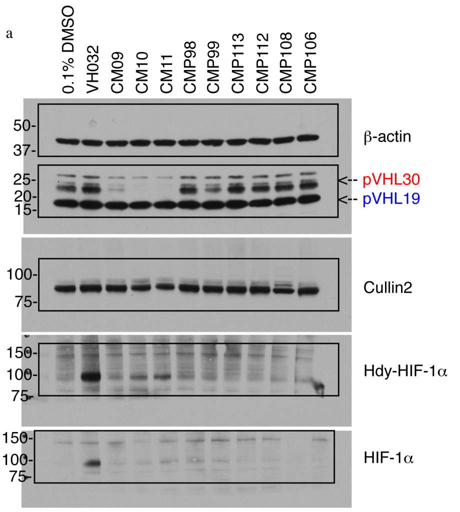

Fig. 4b

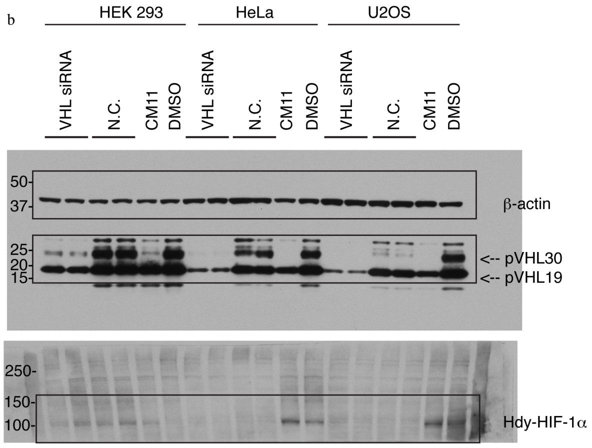

Fig. 5a

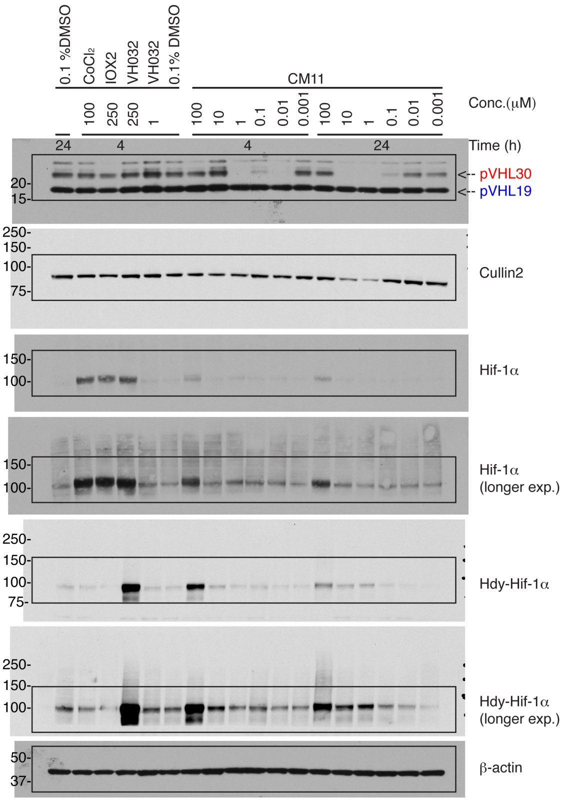

Fig. 5b

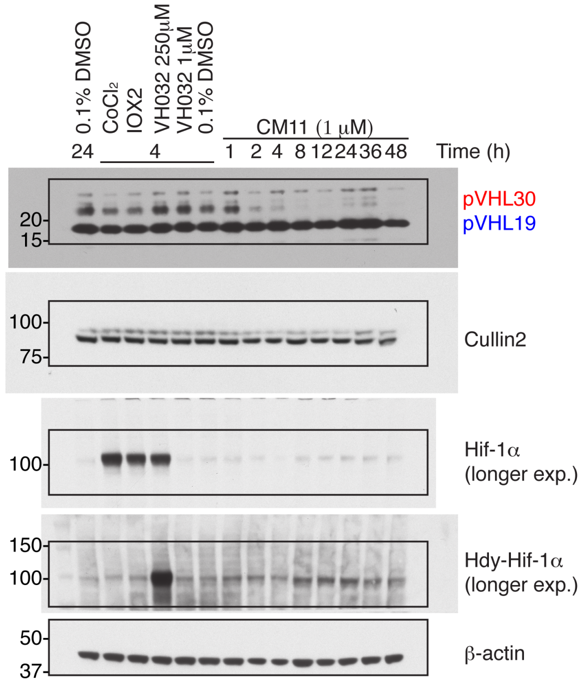

Fig. 6

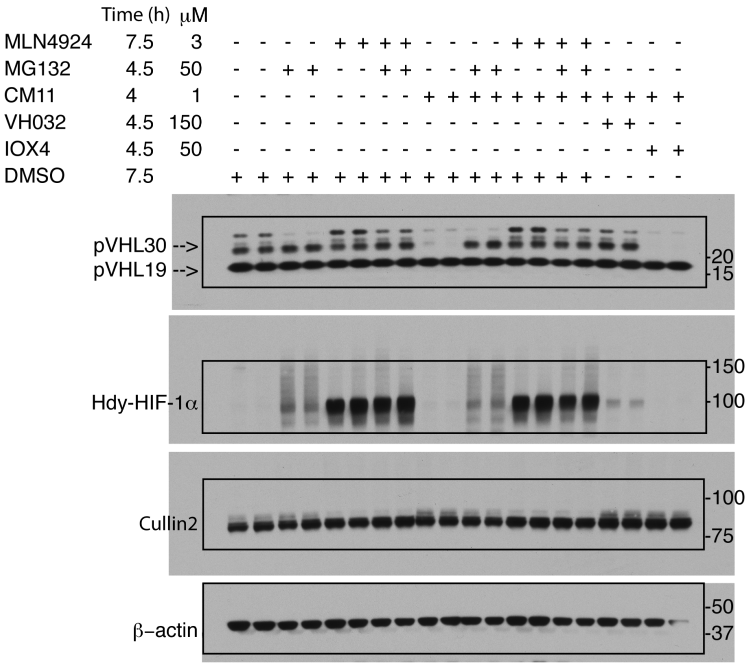

Supplementary Figure 12: Full-scanned images of western-blots shown in the main text figures.

Supplementary Note 1: GENERAL INFORMATION

All chemicals were purchased from commercial vendors and used without further purification, unless indicated otherwise. Reactions were magnetically stirred; commercially available anhydrous solvents were used. All reactions requiring anhydrous conditions were carried out under argon or nitrogen atmosphere using oven-dried glassware. Commercially available anhydrous solvents were used for all reactions. Flash column chromatography was carried out using silica gel (Merck 60 F254 nm). Normal phase TLC was carried out on pre-coated silica plates (Kieselgel 60 F254, BDH) with visualization via UV light (UV 254/365 nm) and/or basic potassium permanganate solution or other suitable stains. Flash column chromatography (FCC) was performed using a Teledyne Isco Combiflash Rf or Rf200i, prepacked columns RediSep Rf Normal Phase Disposable Columns were used. NMR spectra were recorded on a Bruker Ascend 400 or 500 Mz. Chemical shifts are reported in parts per million referenced to residual solvent peaks ($CDCl_3$ = 7.26 ppm). The following abbreviations were used in reporting spectra, s (singlet), d (doublet), t (triplet), q (quartet), m (multiplet), dd (doublet of doublets). Only major rotamer NMR spectra are reported. High Resolution Mass Spectra (HRMS) were recorded on a Bruker microTOF. Low resolution MS and analytical HPLC traces were recorded on an Agilent Technologies 1200 series HPLC connected to an Agilent Technologies 6130 quadrupole LC/MS, connected to an Agilent diode array detector. The column used was a Waters XBridge column (50 mm × 2.1 mm, 3.5 μm particle size), with a gradient from 5 % to 95% of acetonitrile in water (with 0.1 % of formic acid or aqueous ammonia solution) over 3 or 7 minutes. The flow rate was 0.6 mL/min. Preparative HPLC was performed on a Gilson Preparative HPLC System with a Waters XBridge C18 column (100 mm x 19 mm; 5 μm particle size). Details about the conditions for preparative HPLC are provided in the experimental procedures.

Supplementary Note 2: EXPERIMENTAL PROCEDURES

Chemistry

General method A: PEG (1 eq.) was solubilised in dioxane anhydrous (10 ml) and NaH (2 eq.) was added under stirring. The resulting mixture was stirred at r.t. for 3h. The mixture was cooled down to 0 ^{o}C using ice bath and *tert*-butylbromo acetate (2 eq.) was added drop by drop. The resulting mixture was stirred at r.t O/N. The precipitate was filtered off and the organic phase evaporated to dryness. The resulting oil was taken up with ethyl acetate, washed with water, dried over $MgSO_4$ and evaporated to dryness. The resulting oil was purified by column chromatography using a gradient of ethyl acetate from 50% to 100% v/v in heptane.

General method B: *tert*-butyl esters 1, 2, 3 or 12 were dissolved in a solution of 50% v/v trifluroacetic acid in DCM. The resulting solution was stirred for 1 h or until complete conversion of starting material. The solvent was removed under high vacuum. The resulting carboxylic acid was used as crude in the next step without any further purification. To a solution of carboxylic acid in 1 ml DMF were added HATU (1 eq.) and HOBT (1 eq.) and the solution was stirred at room temperature for 5 min. Amine 6, 31 or 32 was added and the pH of the reaction mixture was adjusted to >9 by addition of DIPEA (3 eq.). The mixture was stirred at room temperature until no presence of the starting materials was detected by LC-MS. Water was added and the mixture was extracted with ethyl acetate (×3). The combined organic phases were washed with brine (×2), dried over $MgSO_4$ and evaporated under reduced pressure to give the corresponding crude, which was purified by HPLC using a gradient of 20% to 95% v/v acetonitrile in 0.1% aqueous solution of ammonia to yield the desired compound.

General method C: A mixture of mesilate (1.2 eq.), compound 17 or 18 (2 eq for the synthesis of compound CMP106 and CM108, 1 eq for 22 and 23) and K_2CO_3 (6 eq for the synthesis of CMP106 and CMP108, 3 for 22 and 23) in 1 ml of DMF was stirred O/N at 70 ^{o}C. The reaction mixture was filtered off to afford the crude product, which was purified by HPLC using a gradient of 5% to 95% v/v acetonitrile in 0.1% aqueous solution of formic acid to yield the desired compounds.

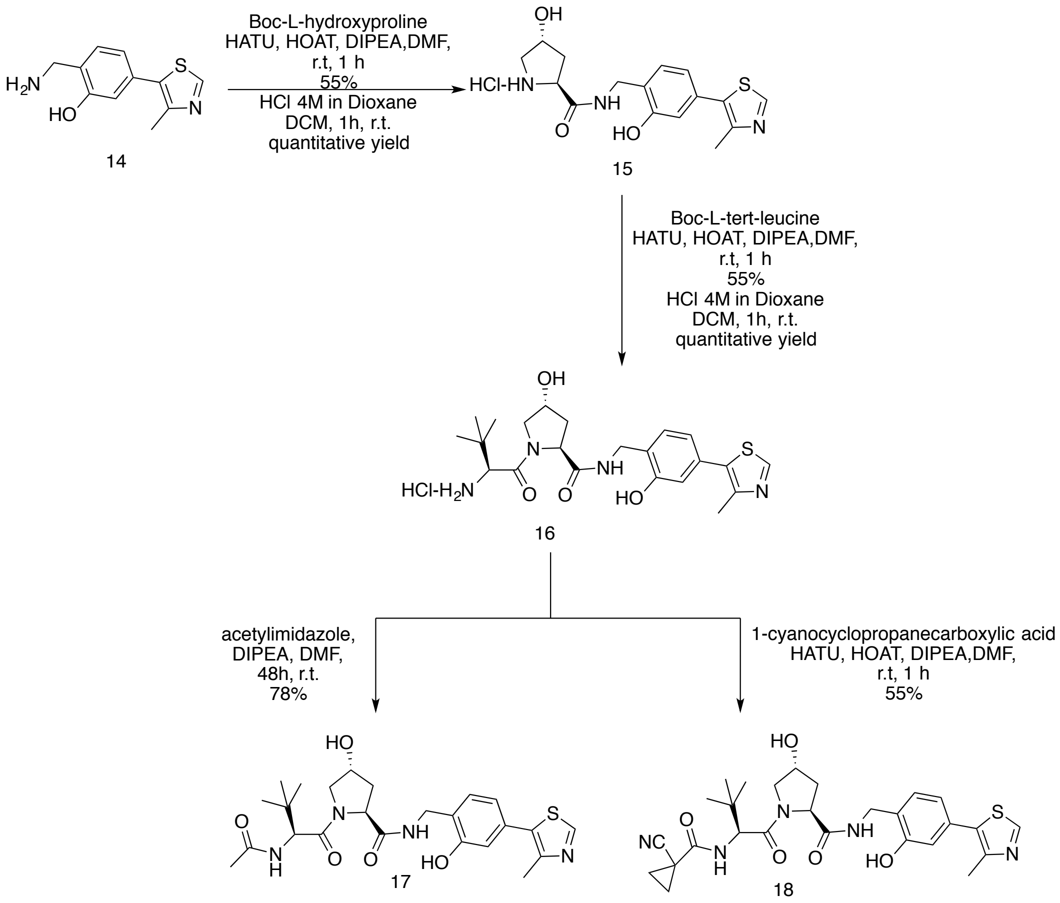

Synthesis of VHL binding moieties 17 and 18.

***(2S,4R)-4-hydroxy-N-(2-hydroxy-4-(4-methylthiazol-5-yl)benzyl)pyrrolidine-2-carboxamide hydrochloride* (15)**

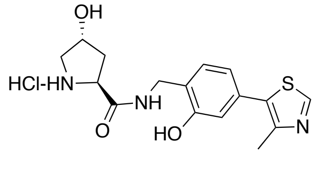

To a solution of *trans*-*N*-(*tert*-Butoxycarbonyl)-4-hydroxy-L-proline (890 mg, 3.84 mmol, 1 eq.) in DMF was added HATU (1.46 g, 3.84 mmol, 1 eq.) and HAOT (522 mg, 3.84 mmol, 1 eq.) and the pH of the reaction mixture was adjusted to >9 by addition of DIPEA (3 eq.). The resulting solution was stirred at room temperature for 5 min. **14** (846 mg, 3.84 mmol, 1 eq.) was added and the mixture was stirred at room temperature until no presence of the starting materials was detected by LC-MS. Water was added and the mixture was extracted with ethyl acetate (×3). The combined organic phases were washed with brine (×2), dried over $MgSO_4$ and evaporated under reduced pressure to give the corresponding crude, which was purified by flash column chromatography using a gradient of 0 to 80% v/v acetone in heptane to yield the titled compound. Yield: 1.298 g, 3 mmol (78%). Analytical data matched those previously reported.[1]

The N-Boc-protected compound was dissolved in 8.31 ml DCM. An equal volume of 4 M HCl in dioxane was added and the reaction mixture stirred at room temperature for 2 h. The solvent was removed under a stream of nitrogen and dried under reduce pressure. The resulting crude was used for the next step without any further purification (quantitative yield). Analytical data matched those previously reported.[1]

***(2S,4R)-1-((S)-2-amino-3,3-dimethylbutanoyl)-4-hydroxy-N-(2-hydroxy-4-(4-methylthiazol-5-yl)benzyl)pyrrolidine-2-carboxamide hydrochloride* (16)**

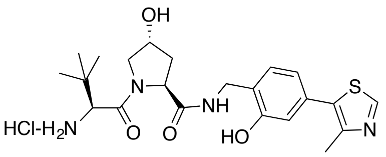

To a solution of *trans-N-*(*tert*-Butoxycarbonyl)-4-hydroxy-L-proline (890 mg, 3.84 mmol, 1 eq.) in DMF was added HATU (1.46 g, 3.84 mmol, 1 eq.) , HAOT (522 mg, 3.84 mmol, 1 eq.) the pH of the reaction mixture was adjusted to >9 by addition of DIPEA (3 eq.) and the solution was stirred at room temperature for 5 min. **14** (846 mg, 3.84 mmol, 1 eq.) was added and the mixture was stirred at room temperature until no presence of the starting materials was detected by LC-MS. Water was added and the mixture was extracted with ethyl acetate (×3). The combined organic phases were washed with brine (×2), dried over $MgSO_4$ and evaporated under reduced pressure to give the corresponding crude, which was purified by flash column chromatography using a gradient of 0 to 80% v/v acetone in heptane to yield the titled compound. Yield: 1.915 g, 3.61 mmol (94%). Analytical data matched those previously reported.[1]

The N-Boc-protected compound was dissolved in 10 ml DCM. An equal volume of 4 M HCl in dioxane was added and the reaction mixture stirred at room temperature for 2 h. The solvent was removed under a stream of nitrogen and dried under reduce pressure. The resulting crude was used for the next step without any further purification (quantitative yield). Analytical data matched those previously reported.[1]

***(2S,4R)-1-((S)-2-acetamido-3,3-dimethylbutanoyl)-4-hydroxy-N-(2-hydroxy-4-(4-methylthiazol-5-yl)benzyl)pyrrolidine-2-carboxamide* (17)**

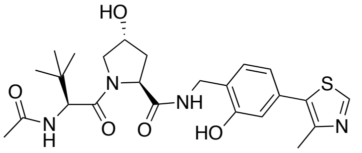

The amine precursor **16** (100,7 mg, 0.240 mmol, 1 eq.) was dissolved in 1 ml of DMF, acetylimidazole (31.7 mg, 0.288 mmol, 1.2 eq) and DIPEA (0.090 ml, 0.48 mmol, 2 eq.) were added to the solution. After stirring the mixture for 48h at room temperature, the solvent was evaporated under reduced pressure to give the corresponding crude, which was purified by HPLC using a gradient of 5% to 95% v/v acetonitrile in 0.1% aqueous solution of formic acid to yield the titled compound. Yield: 91 mg, 0.187 mmol (78%). **^{1}H NMR** (400 MHz, $CDCl_3$): δ 9.25 (s, 1H), 8.70 (s, 1H), 7.97 (t, J=6.5 Hz, 1H), 7.15 (d, J=7.5 Hz, 1H), 6.83 - 6.80 (m, 2H), 6.72 (d, J=8.8 Hz, 1H), 4.92 - 4.88 (m, 1H), 4.57 (s, 1H), 4.52 - 4.42 (m, 2H), 4.26 - 4.14 (m, 2H), 3.59 (dd, J=2.9, 11.1 Hz, 1H), 2.53 - 2.45 (m, 4H), 2.24 - 2.17 (m, 1H), 1.85 (s, 3H), 0.83 (s, 9H); **^{13}C NMR** (101 MHz, $CDCl_3$): δ 171.8, 171.2, 155.9, 150.7, 148.1, 132.8, 131.7, 131.0, 124.2, 120.6, 117.1, 70.3, 58.1, 57.7, 57.1, 39.8, 35.5, 34.8, 26.3, 22.6, 16.0. **MS (ESI)** m/z: $[M+H]^+$ calculated for: $C_{24}H_{32}N_4O_5S$: 488.21; observed: 484.3.

***(2S,4R)-1-((S)-2-(1-cyanocyclopropane-1-carboxamido)-3,3-dimethylbutanoyl)-4-hydroxy-N-(2-hydroxy-4-(4-methylthiazol-5-yl)benzyl)pyrrolidine-2-carboxamide* (18)**

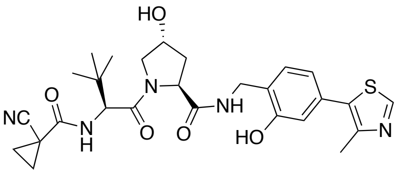

1-cyanocyclopropanecarboxylic acid (69 mg, 0.62 mmol, 1 eq.) was solubilized in 3 ml of DMF. HATU (235 mg, 0.62 mmol, 1 eq.) and HOAT (84.4 mg, 0.62 mmol, 1 eq.) were added and the resulting mixture was stirred at r.t. for 5 min. The amine precursor of **16** (300 mg, 0.62 mmol, 1 eq.) was added and the pH was adjusted to pH>9 using DIPEA (400 mg, 0.5 ml, 3.1 mmol, 5 eq.). The resulting mixture was stirred at r.t. until complete conversion of the starting material. Water was added, and the mixture was extracted with ethyl acetate (×3). The combined organic phases were washed with brine (×1), dried over MgSO4, and evaporated to afford the corresponding crude compound that was purified by flash column chromatography using a gradient of 10% to 70% acetone in heptane to yield the title compound as a white solid. Yield: 200 mg, 0.37 mmol (60%). **^{1}H NMR** (400 MHz, $CDCl_3$): δ 9.29 (s, 1H), 8.65 (s, 1H), 8.02 (t, J=6.4 Hz, 1H), 7.12 (d, J=7.7 Hz, 1H,), 6.99 (d, J=8.0 Hz, 1H), 6.94 (d, J=1.8 Hz, 1H), 6.86 (dd, J=1.8, 7.7 Hz, 1H), 4.72 (t, J=8.0 Hz, 1H), 4.54 (s, 1H), 4.44 - 4.35 (m, 2H), 4.19 (dd, J=5.5, 14.6 Hz, 1H), 3.87 (d, J=11.0 Hz, 1H), 3.62 (dd, J=3.7, 11.0 Hz, 1H), 3.50 (s, 1H), 2.49 (s, 3H), 2.43 - 2.37 (m, 1H), 2.13 - 2.06 (m, 1H), 1.66 - 1.37 (m, 4H), 0.89 (s, 8H); **^{13}C NMR** (101 MHz, $CDCl_3$): δ 172.8, 170.8, 165.8, 155.8, 150.5, 148.3, 133.3, 131.6, 131.2, 123.9, 120.9, 119.6, 118.2, 70.1, 58.6, 58.3, 56.7, 55.7, 40.0, 35.7, 26.2, 18.6, 17.9, 17.8, 17.2, 16.1, 13.8. **MS (ESI)** m/z: $[M+H]^+$ calculated for: $C_{27}H_{33}N_5O_5S$: 539.22; observed: 540.3.

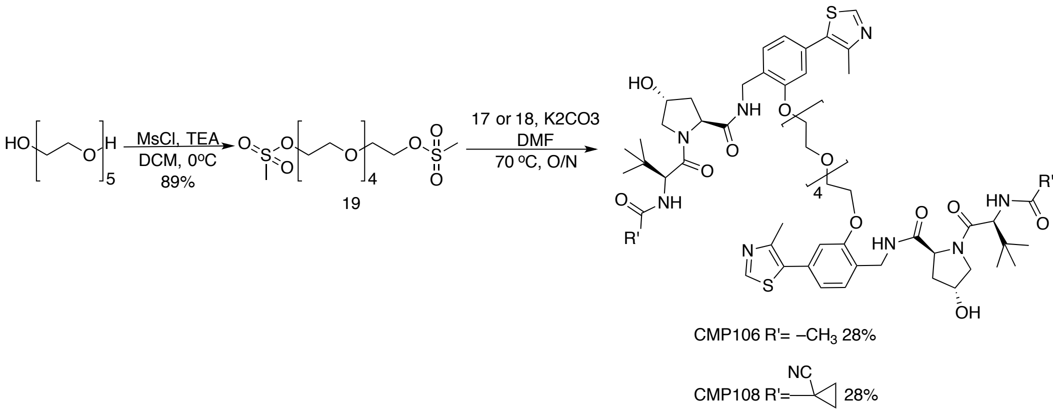

Synthesis of Homo-PROTACs CMP106 and CMP108 symmetrically derivatized from the phenyl group.

(2S,2'S,4R,4'R)-N,N'-((((3,6,9,12-tetraoxatetradecane-1,14-diyl)bis(oxy))bis(4-(4-methylthiazol-5-yl)-2,1-phenylene))bis(methylene))bis(1-((S)-2-acetamido-3,3-dimethylbutanoyl)-4-hydroxypyrrolidine-2-carboxamide) **(CMP106)**

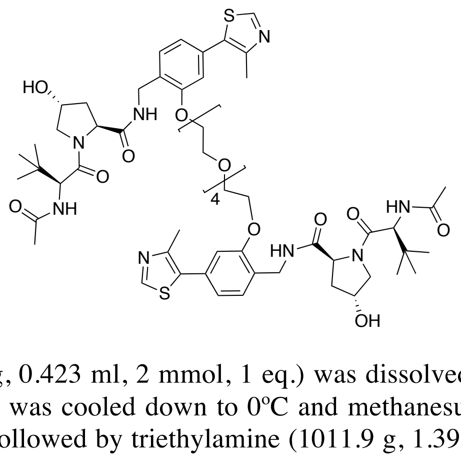

Pentaethylene glycol (476.56 mg, 0.423 ml, 2 mmol, 1 eq.) was dissolved in 4 ml of dry DCM. The temperature of the resulting mixture was cooled down to 0ºC and methanesulfonyl chloride (687.3mg, 0.464 ml, 16 mmol, 3 eq.) was added followed by triethylamine (1011.9 g, 1.39 ml, 10 mmol, 5 eq.). The resulting mixture was stirred at 0 ºC for 4 h. A 10% aqueous solution of $NaHSO_4$ was added till pH=3. The

aqueous phase was extracted with DCM (x5). The organic phases were combined, dried over $MgSO_4$ and concentrated in vacuum to afford the title compound as an orange oil. Yield: 701 mg, 1.77 mmol (89%). **^{1}H NMR** (400 MHz, $CDCl_3$): δ 4.33 - 4.30 (m, 4H), 3.72 - 3.69 (m, 4H), 3.62 - 3.56 (m, 12H), 3.02 (s, 6H).[2,3]

Following general method C**,** from **17** (24.3 mg, 0.05 mmol, 2 eq.), **19** (11.83 mg, 0.03 mmol, 1.2 eq.) and and K_2CO_3 (41.46 mg, 0.3 mmol, 6 eq.), the title compound was obtained as a white solid. Yield: 7.8 mg, 0.006 mmol (26%). **^{1}H NMR** (400 MHz, $CDCl_3$): δ 8.60 (s, 2H), 7.39 - 7.35 (m, 2H), 7.26 (d, *J*=7.6 Hz, 2H), 6.91 - 6.88 (m, 2H), 6.83 - 6.80 (m, 2H), 6.36 - 6.13 (m, 2H), 4.60 - 4.32 (m, 10H), 4.18 - 4.05 (m, 4H), 3.97 - 3.79 (m, 6H), 3.71 - 3.54 (m, 18H), 2.44 (s, 6H), 2.17 - 1.86 (m, 8H), 0.87 (s, 18H); **^{13}C NMR** (101 MHz, $CDCl_3$): δ 171.3, 171.1, 171.0, 170.7, 170.5, 156.8, 156.8, 150.3, 148.5, 132.2, 131.7, 130.0, 129.8, 127.1, 126.9, 122.1, 122.0, 112.8, 112.8, 71.3, 70.7, 70.6, 70.5, 70.5, 70.5, 70.4 , 70.2, 70.1, 69.7, 67.9, 58.9, 58.6, 57.6, 57.5, 56.9, 56.7, 42.7, 39.1, 39.0, 37.1, 36.4, 35.4, 35.1, 26.4, 26.4, 23.2, 23.1, 16.2. HR**MS (ESI)** m/z: $[M+H]^+$ calculated for: $C_{58}H_{82}N_8O_{14}S_2$: 1178.54; observed: 1179.6422.

(2S,2'S,4R,4'R)-N,N'-((((3,6,9,12-tetraoxatetradecane-1,14-diyl)bis(oxy))bis(4-(4-methylthiazol-5-yl)-2,1-phenylene))bis(methylene))bis(1-((S)-2-(1-cyanocyclopropane-1-carboxamido)-3,3-dimethylbutanoyl)-4-hydroxypyrrolidine-2-carboxamide) **(CMP108)**

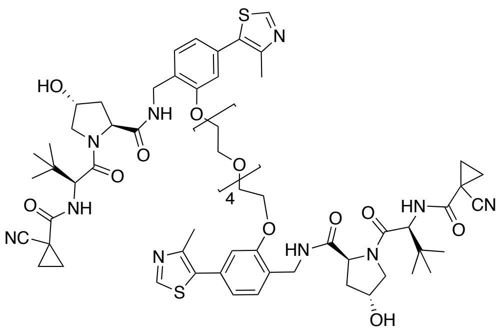

Pentaethylene glycol (476.56 mg, 0.423 ml, 2 mmol, 1 eq.) was dissolved in 4 ml of dry DCM. The temperature of the resulting mixture was cooled down to 0ºC and methanesulfonyl chloride (687.3mg, 0.464 ml, 16 mmol, 3 eq.) was added followed by triethylamine (1011.9 g, 1.39 ml, 10 mmol, 5 eq.). The resulting mixture was stirred at 0 ºC for 4 h. A 10% aqueous solution of $NaHSO_4$ was added till pH=3. The aqueous phase was extracted with DCM (x5). The organic phases were combined, dried over $MgSO_4$ and concentrated in vacuum to afford the title compound as a orange oil. Yield: 701 mg, 1.77 mmol (89%). **^{1}H NMR** (400 MHz, $CDCl_3$): δ 4.33 - 4.30 (m, 4H), 3.72 - 3.69 (m, 4H), 3.62 - 3.56 (m, 12H), 3.02 (s, 6H).[2,3]

Following general method C, from **18** (27 mg, 0.05 mmol, 2 eq.), **19** (11.83 mg, 0.03 mmol, 1.2 eq.) and K_2CO_3 (41.46 mg, 0.3 mmol, 6 eq.), the titled compound was obtained as a white solid. Yield: 9.1 mg, 0.007 mmol (28%). **^{1}H NMR** (400 MHz, $CDCl_3$): δ 8.61 (s, 2H), 7.41 - 7.38 (m, 2H), 7.26 (d, *J*=8.1 Hz, 2H), 7.00 (d, *J*=8.1 Hz, 2H), 6.91 - 6.88 (m, 2H), 6.85 - 6.81 (m, 2H), 4.57 - 4.52 (m, 2H), 4.44 - 4.36 (m, 8H), 4.19 - 4.08 (m, 4H), 3.89 - 3.53 (m, 22H), 2.45 (s, 6H), 2.24 - 2.17 (m, 2H), 2.08 - 2.02 (m, 2H), 1.61 - 1.37 (m, 8H), 0.88 (s, 18H); **^{13}C NMR** (101 MHz, $CDCl_3$): δ 170.9, 170.0, 165.4, 156.9, 150.4, 148.5, 132.3, 131.7, 130.0, 126.9, 122.0, 119.6, 112.9, 70.7, 70.41, 70.38, 70.2, 69.6, 67.9, 58.9, 58.4, 56.6, 39.2, 37.0, 36.0, 26.3, 17.9, 17.7, 16.2, 13.7. **HRMS (ESI)** m/z: $[M+H]^+$ calculated for: $C_{64}H_{84}N_{10}O_{14}S_2$: 1280.56; observed: 1281.6661.

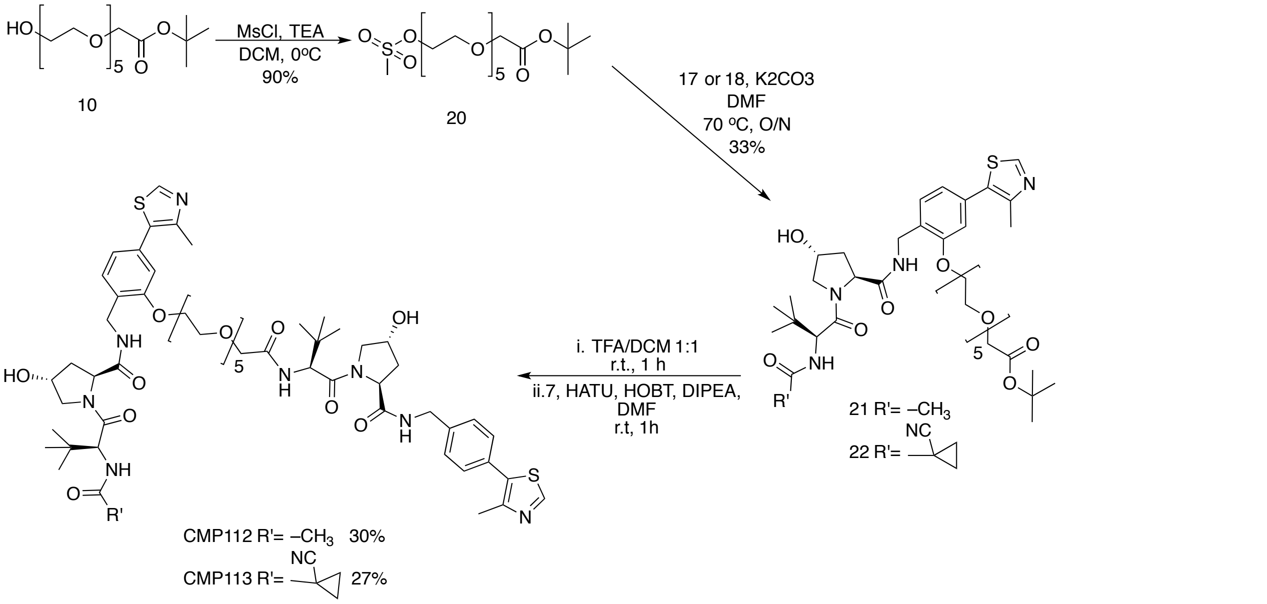

Synthesis of asymmetric Homo-PROTACs CMP112 and CMP113.

tert-butyl 17-((methylsulfonyl)oxy)-3,6,9,12,15-pentaoxaheptadecanoate (20)

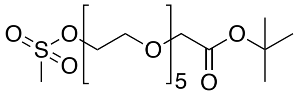

10 (251 mg, 0.712 mmol, 1 eq.) was dissolved in 1.4 ml of dry DCM. The temperature of the resulting mixture was cooled down to 0ºC and methanesulfonyl chloride (122.3 mg, 0.082 ml, 1.068 mmol, 1.5 eq.) was added followed by triethylamine (216.14 mg, 0.3 ml, 2.136 ml, 3 eq.). The resulting mixture was stirred at 0 ºC for 4 h. A 10% aqueous solution of $NaHSO_4$ was added till pH=3. The aqueous phase was extracted with DCM (x5). The organic phases were combined, dried over $MgSO_4$ and concentrated in vacuum to afford the title compound as a orange oil. Yield: 276 mg, 0.641 mmol (90%). **^{1}H NMR** (400 MHz, $CDCl_3$): δ 4.32 - 4.30 (m, 2H), 3.95 (s, 2H), 3.71 - 3.57 (m, 18H), 3.02 (s, 3H, s), 1.41 (s, 9H). **^{13}C NMR** (101 MHz, $CDCl_3$): δ 169.7, 81.5, 70.72, 70.65, 70.61, 70.58, 70.5, 69.3, 69.0, 37.7, 28.1.

***tert-butyl 17-(2-(((2S,4R)-1-((S)-2-acetamido-3,3-dimethylbutanoyl)-4-hydroxypyrrolidine-2-carboxamido)methyl)-5-(4-methylthiazol-5-yl)phenoxy)-3,6,9,12,15-pentaoxaheptadecanoate* (21)**

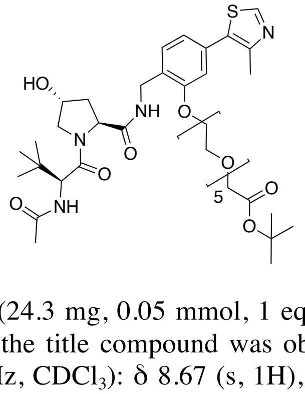

Following general method C**,** from **17** (24.3 mg, 0.05 mmol, 1 eq.), **20** (26 mg, 0.06 mmol, 1.2 eq.) and K_2CO_3 (20.73 mg, 0.15 mmol, 3 eq.), the title compound was obtained as a white solid. Yield: 17 mg, 0.021 mmol (33%). **^{1}H NMR** (400 MHz, $CDCl_3$): δ 8.67 (s, 1H), 7.32 (d, J = 7.8 Hz, 2H), 6.95 (dd, J =

1.6, 7.6 Hz, 1H,), 6.88 (d, J = 1.8 Hz, 1H), 4.65 - 4.60 (m, 1H), 4.53 - 4.43 (m, 2H), 4.39 - 4.36 (m, 1H), 4.24 - 4.13 (m, 2H), 4.00 (d, J = 7.0 Hz, 2H), 3.92 - 3.87 (m, 2H), 3.77 - 3.59 (m, 20H), 3.08 (s, 2H), 2.51 (s, 3H), 2.38 - 2.31 (m, 1H), 1.98 (s, 3H).**^{13}C NMR** (101 MHz, $CDCl_3$): δ 171.2, 170.8, 170.4, 169.7, 156.8, 150.3, 148.5, 132.2, 131.7, 129.8, 126.9, 122.0, 112.8, 81.6, 70.8, 70.71, 70.69, 70.60, 70.57, 70.55, 70.52, 70.49, 70.47, 70.1, 69.6, 69.3, 69.02, 68.98, 67.9, 58.6, 57.5, 56.7, 39.0, 37.7, 36.5, 35.2, 28.1, 26.4, 23.2, 16.1. **HRMS (ESI)** m/z: $[M+H]^+$ calculated for: $C_{40}H_{62}N_4O_{12}S$: 822.41; observed: 823.4828.

tert-butyl(14-(2-(((2S,4R)-1-((S)-2-(1-cyanocyclopropane-1-carboxamido)-3,3-dimethylbutanoyl)-4-hydroxypyrrolidine-2-carboxamido)methyl)-5-(4-methylthiazol-5-yl)phenoxy)-3,6,9,12-tetraoxatetradecyl) carbonate **(22)**

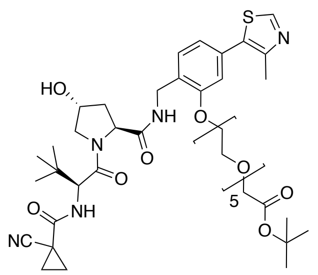

Following general method C, from **18** (27 mg, 0.05 mmol, 1 eq.), **20** (26 mg, 0.06 mmol, 1.2 eq.) and K_2CO_3 (20.73 mg, 0.15 mmol, 3 eq.), the title compound was obtained as a white solid. Yield: 17 mg, 0.02 mmol (33%). **^{1}H NMR** (400 MHz, $CDCl_3$): δ 8.61 (s, 1H), 7.33 - 7.25 (m, 2H), 6.97 (d, *J*=9.1 Hz, 1H), 6.92 - 6.89 (m, 1H), 6.84 (d, *J*=1.5 Hz, 1H), 4.59 - 4.55 (m, 1H), 4.45 - 4.38 (m, 4H), 4.22 - 4.10 (m, 2H), 3.93 - 3.54 (m, 24H), 2.46 (s, 3H), 2.32 - 2.24 (m, 1H), 2.10 - 2.04 (m, 1H), 1.63 - 1.52 (m, 2H), 1.45 - 1.39 (m, 12H), 0.87 (s, 9H); **^{13}C NMR** (101 MHz, $CDCl_3$): δ 170.6, 170.1, 169.7, 165.4, 156.9, 150.3, 148.5, 132.3, 131.7, 130.0, 126.9, 122.0, 119.7, 112.9, 81.7, 70.72, 70.66, 70.5, 70.4, 70.3, 69.6, 69.0, 68.0, 58.8, 58.4, 56.6, 39.3, 36.7, 35.8, 28.1, 26.3, 17.8, 16.2, 13.7. **HRMS (ESI)** m/z: $[M+H]^+$ calculated for: $C_{43}H_{63}N_5O_{12}S$: 873.42; observed: 874.4934.

(2S,4R)-1-((S)-2-acetamido-3,3-dimethylbutanoyl)-4-hydroxy-N-(2-(((S)-19-((2S,4R)-4-hydroxy-2-((4-(4-methylthiazol-5-yl)benzyl)carbamoyl)pyrrolidine-1-carbonyl)-20,20-dimethyl-17-oxo-3,6,9,12,15-pentaoxa-18-azahenicosyl)oxy)-4-(4-methylthiazol-5-yl)benzyl)pyrrolidine-2-carboxamide **(CMP112)**

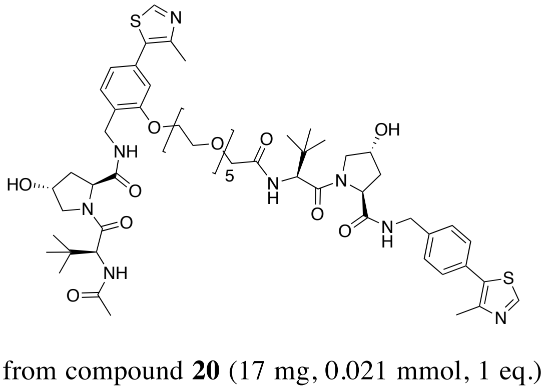

Following general method B, from compound **20** (17 mg, 0.021 mmol, 1 eq.) and trifluoroacetic acid (0.5 ml in 0.5 ml of DCM), the carboxylic acid derivative or 38 was obtained as an oil. Yield: 13 mg, 0.017 mmol (quantitative). **MS (ESI)** m/z: $[M+H]^+$ calculated for: $C_{36}H_{54}N_4O_{12}S$: 766.35; observed: 767.4.

From the carboxylic acid (13 mg, 0.017 mmol, 1 eq.) in 0.5 ml DMF, HATU (6.49 mg, 0.017 mmol, 1 eq.), HOAT (2.31 mg, 0.017 mmol, 1 eq.), compound **7** (7.90 mg, 0.017 mmol, 1 eq.) and DIPEA (3 eq.). The titled compound was obtained as a white solid. Yield: 6 mg, 0.005 mmol (30%). **^{1}H NMR** (400 MHz, $CDCl_3$): δ 8.61 (s, 2H), 7.49 - 7.45 (m, 1H), 7.32 - 7.24 (m, 6H), 6.90 - 6.87 (m, 1H), 6.79 (d, *J*=2.4 Hz,

1H), 6.24 (d, *J*=8.9 Hz, 1H), 4.61 - 4.29 (m, 10H), 4.11 - 3.52 (m, 27H), 2.44 (s, 6H), 2.30 (t, *J*=13.3 Hz, 1H), 2.18 - 2.03 (m, 3H), 0.87 (s, 9H); **^{13}C NMR** (101 MHz, $CDCl_3$): δ 171.2, 171.1, 170.7, 170.4, 156.7, 150.3, 148.4, 138.3, 132.2, 130.9, 129.7, 129.4, 128.0, 127.0, 122.0, 112.8, 70.9, 70.6, 70.5, 70.4, 70.3, 70.2, 69.6, 67.9, 59.0, 58.8, 57.7, 57.1, 43.1, 39.0, 37.1, 36.8, 35.6, 35.5, 26.42, 26.38, 23.0, 16.13, 16.06. HR**MS (ESI)** m/z: $[M+H]^+$ calculated for: $C_{58}H_{82}N_8O_{14}S_2$: 1178.54; observed: 1179.6608.

***(2S,4R)-1-((S)-2-(tert-butyl)-20-(2-(((2S,4R)-1-((S)-2-(1-cyanocyclopropane-1-carboxamido)-3,3-dimethylbutanoyl)-4-hydroxypyrrolidine-2-carboxamido)methyl)-5-(4-methylthiazol-5-yl)phenoxy)-4-oxo-6,9,12,15,18-pentaoxa-3-azaicosanoyl)-4-hydroxy-N-(4-(4-methylthiazol-5-yl)benzyl)pyrrolidine-2-carboxamide* (CMP113)**

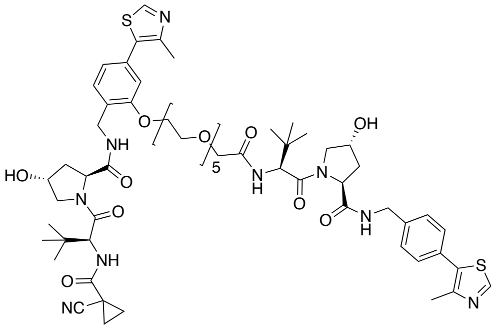

Following general method B, from compound **20** (17 mg, 0.02 mmol, 1 eq.) and trifluoroacetic acid (0.5 ml in 0.5 ml of DCM), the carboxylic acid derivative was obtained as an oil. Yield: 15.7 mg, 0.019 mmol (quantitative). **MS (ESI)** m/z: $[M+H]^+$ calculated for: $C_{39}H_{55}N_5O_{12}S$: 817.36; observed: 818.4.

From the obtained carboxylic acid (15.7 mg, 0.019 mmol, 1 eq.) in 0.5 ml DMF, HATU (7.22 mg, 0.019 mmol, 1 eq.), HOAT (2.58 mg, 0.019 mmol, 1 eq.), compound **7** (8.73 mg, 0.019 mmol, 1 eq.) and DIPEA (3 eq.), the final compound was isolated as white solid. Yield: 6.3 mg, 0.005 mmol (27%). **^{1}H NMR** (400 MHz, $CDCl_3$): δ 8.61 (s, 2H), 7.58 - 7.54 (m, 1H), 7.31 - 7.25 (m, 5H), 7.02 (d, *J*=9.7 Hz, 1H), 6.88 - 6.85 (m, 1H), 6.78 (d, *J*=1.5 Hz, 1H), 4.59 - 4.56 (m, 2H), 4.47 - 4.25 (m, 6H), 4.13 - 3.52 (m, 20H), 2.47 - 2.42 (m, 6H), 2.34 - 2.27 (m, 1H), 2.19 - 2.03 (m, 5H), 1.63 - 1.52 (m, 2H), 1.48 - 1.37 (m, 2H), 0.90 (s, 18H); **^{13}C NMR** (101 MHz, $CDCl_3$): δ 171.2, 171.1, 170.7, 170.3, 165.4, 156.6, 150.3, 148.4, 148.3, 138.3, 132.0, 131.8, 131.7, 130.7, 129.5, 129.4, 127.9, 126.9, 122.0, 119.7, 112.6, 71.0, 70.7, 70.5, 70.4, 70.32, 70.28, 70.25, 69.6, 67.9, 59.1, 58.8, 58.5, 57.3, 57.1, 56.7, 43.1, 39.0, 37.3, 36.8, 36.2, 35.4, 26.4, 26.3, 17.9, 17.7, 16.1, 16.0, 13.7. **HRMS (ESI)** m/z: $[M+H]^+$ calculated for: $C_{61}H_{83}N_9O_{14}S_2$: 1229.55; observed: 1230.6650.

Supplementary Note 3: NMR SPECTRA of Homo-PROTACs and intermediates

CM09, ^{1}H-NMR and ^{13}C-NMR in $CDCl_3$

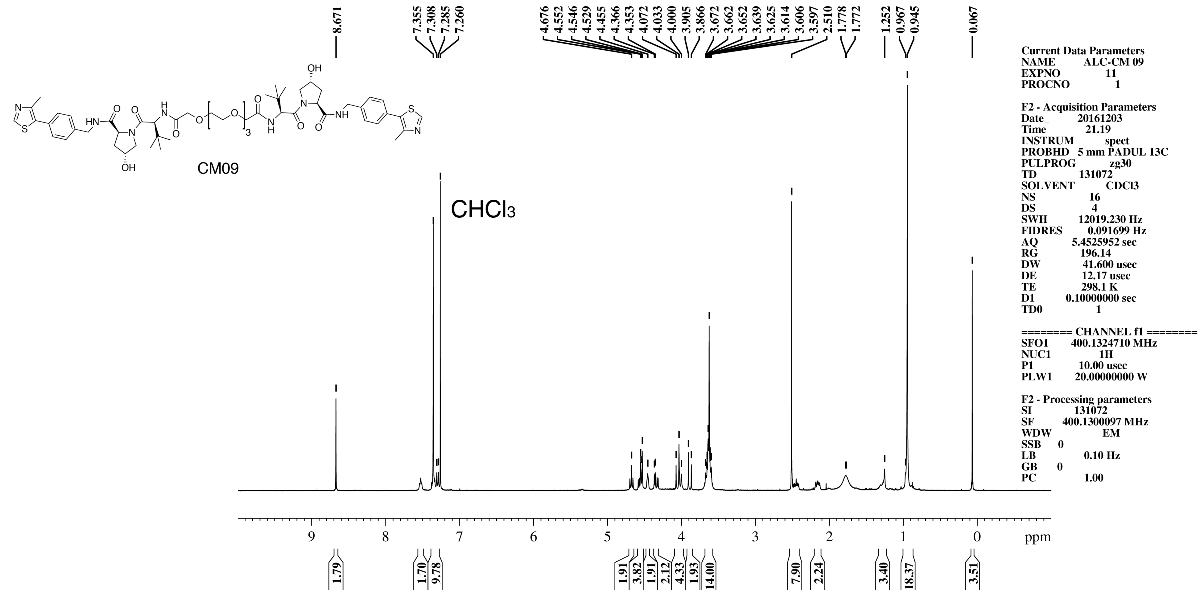

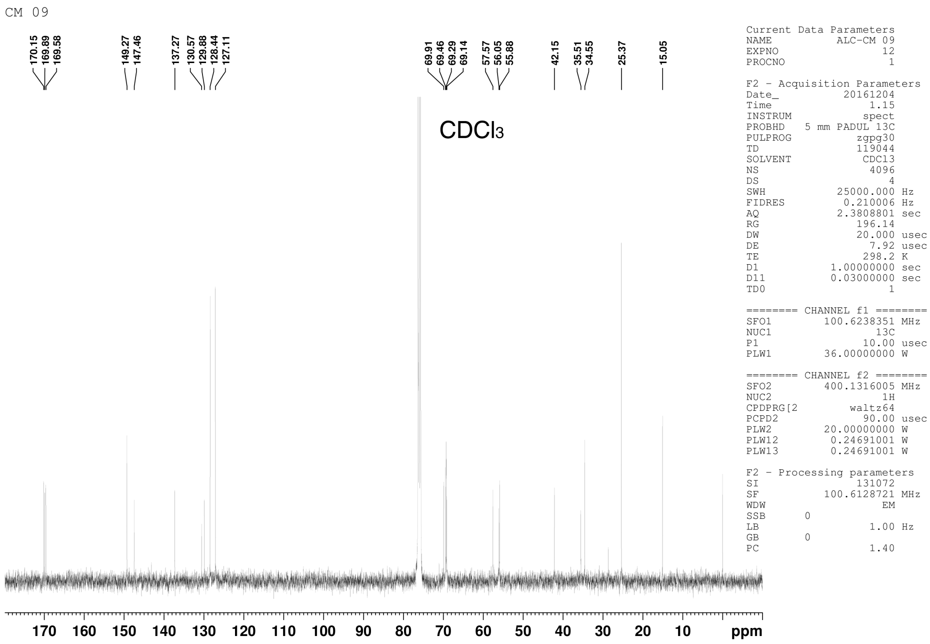

CM10, ^{1}H-NMR and ^{13}C-NMR in $CDCl_3$

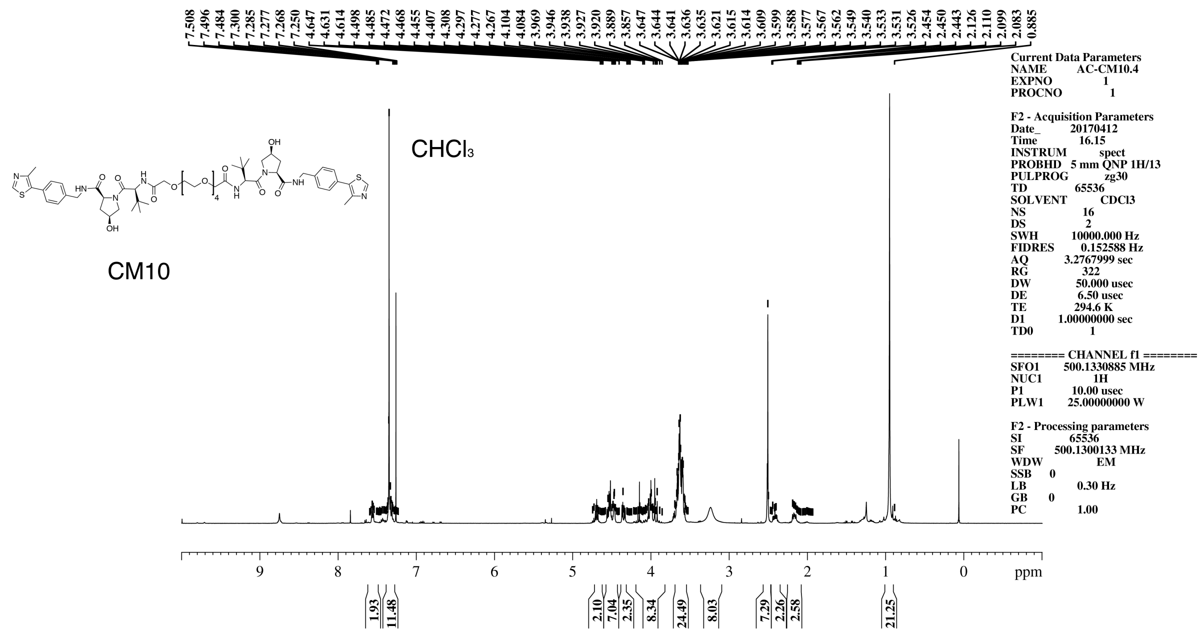

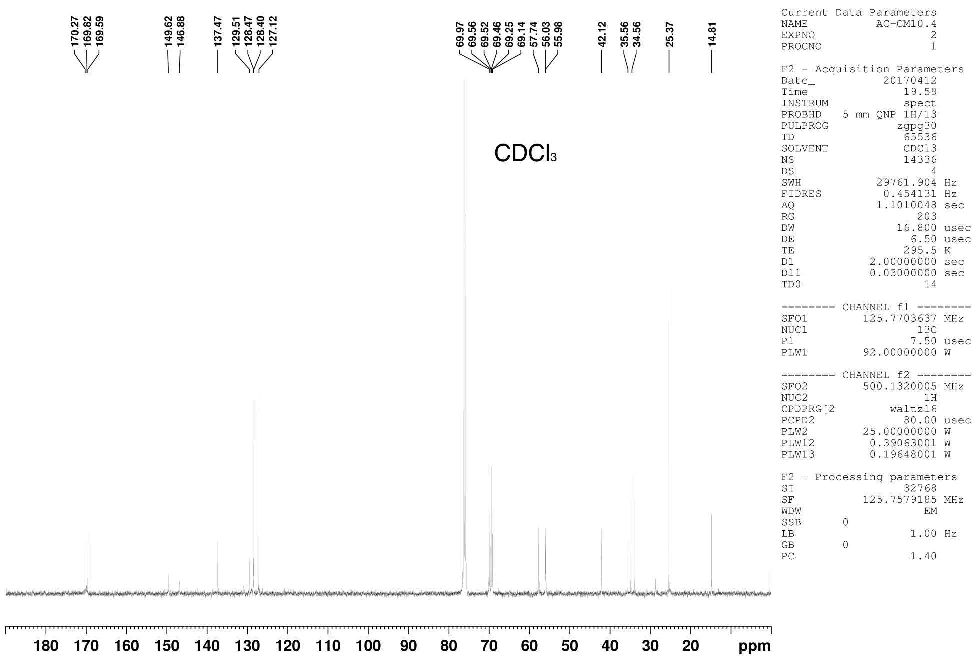

CM11, ^{1}H-NMR and ^{13}C-NMR in $CDCl_3$

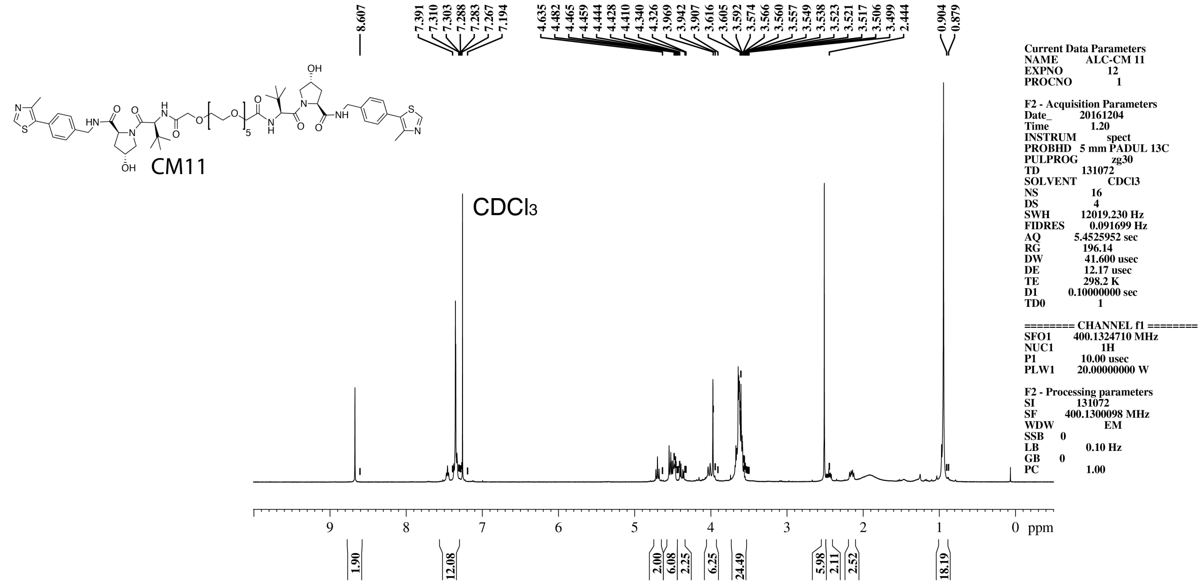

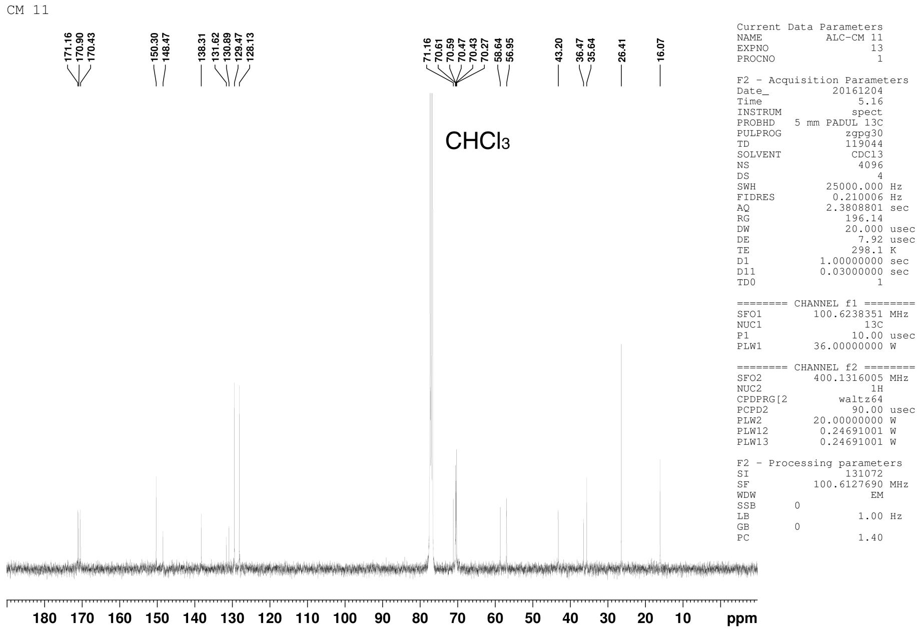

9, ^{1}H-NMR and ^{13}C-NMR in $CDCl_3$

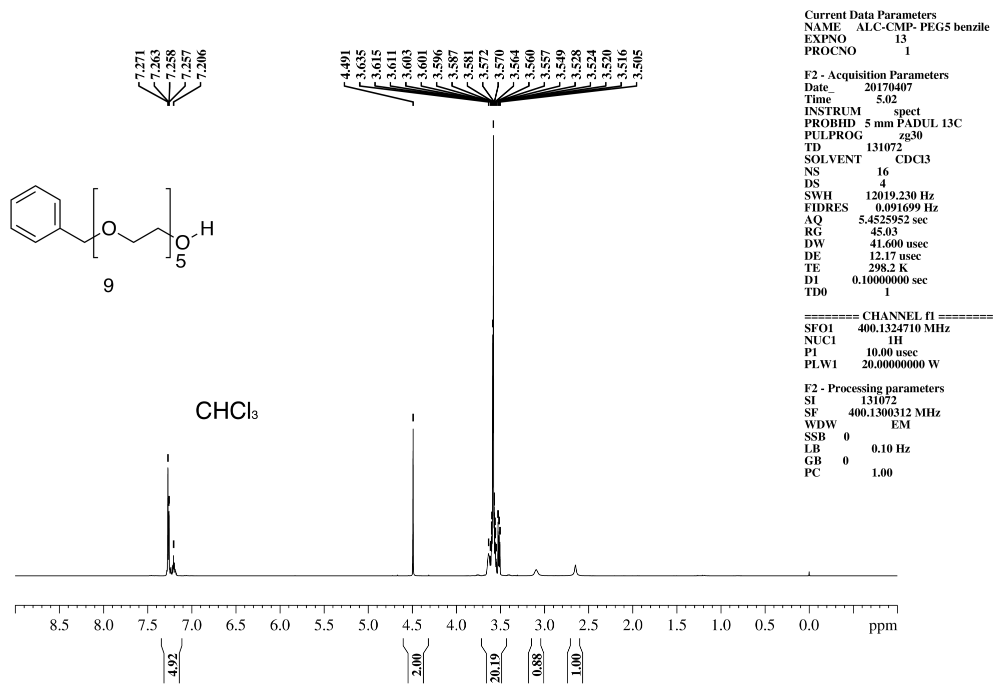

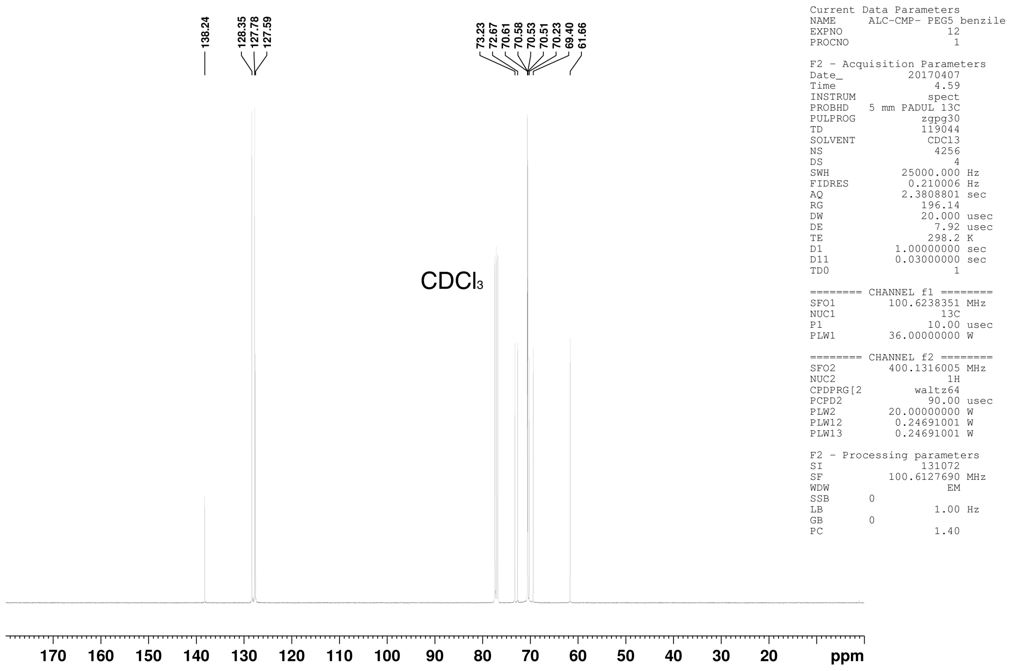

10, ^{1}H-NMR and ^{13}C-NMR in $CDCl_3$

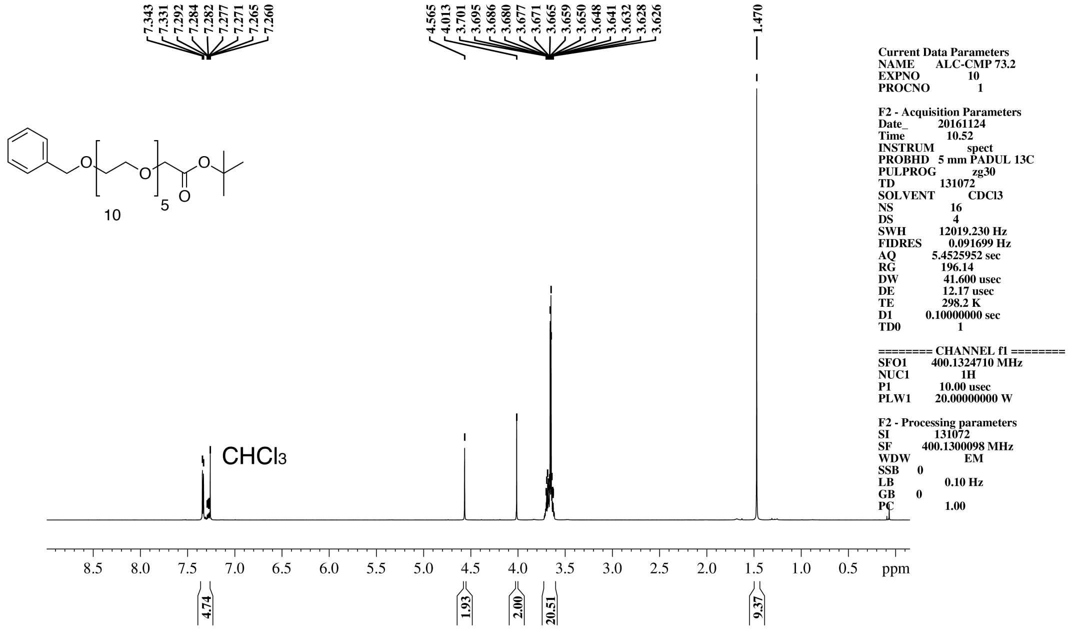

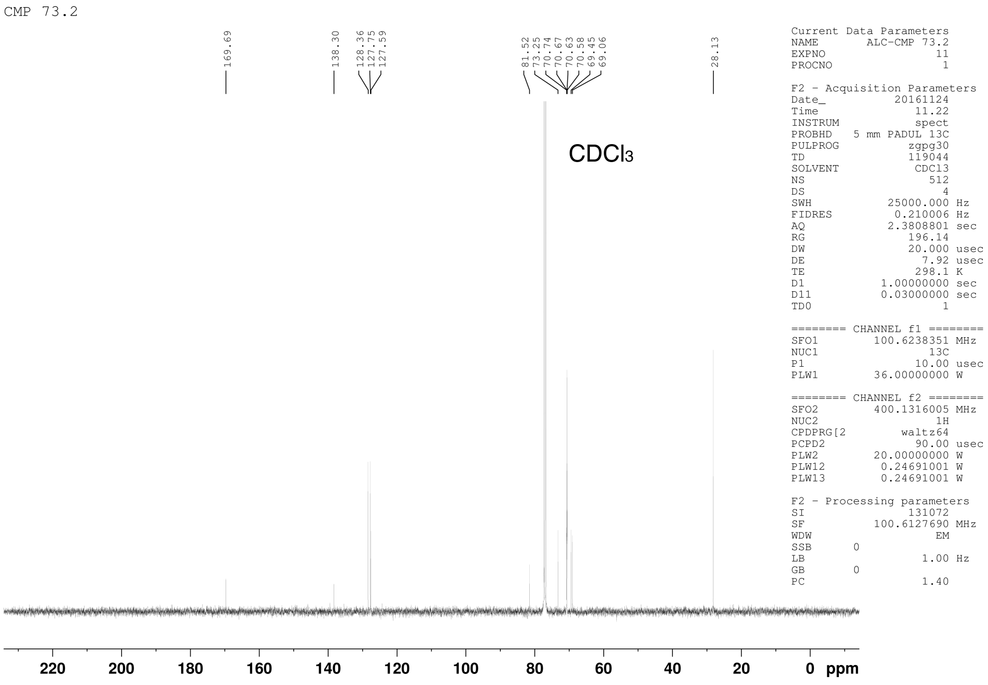

11, ^{1}H-NMR and ^{13}C-NMR in $CDCl_3$

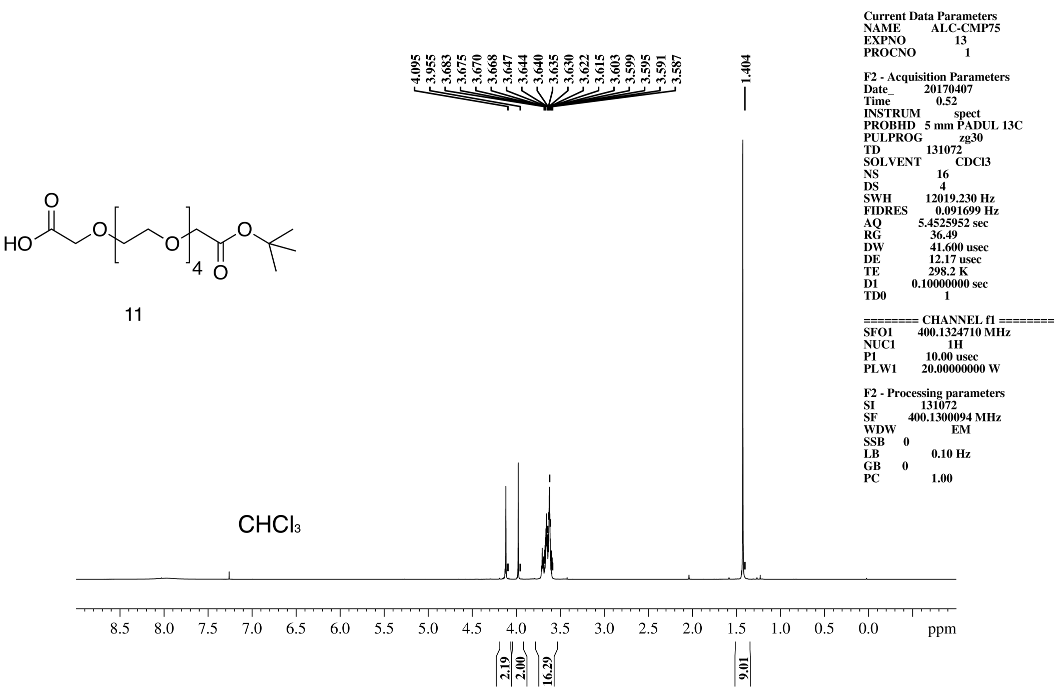

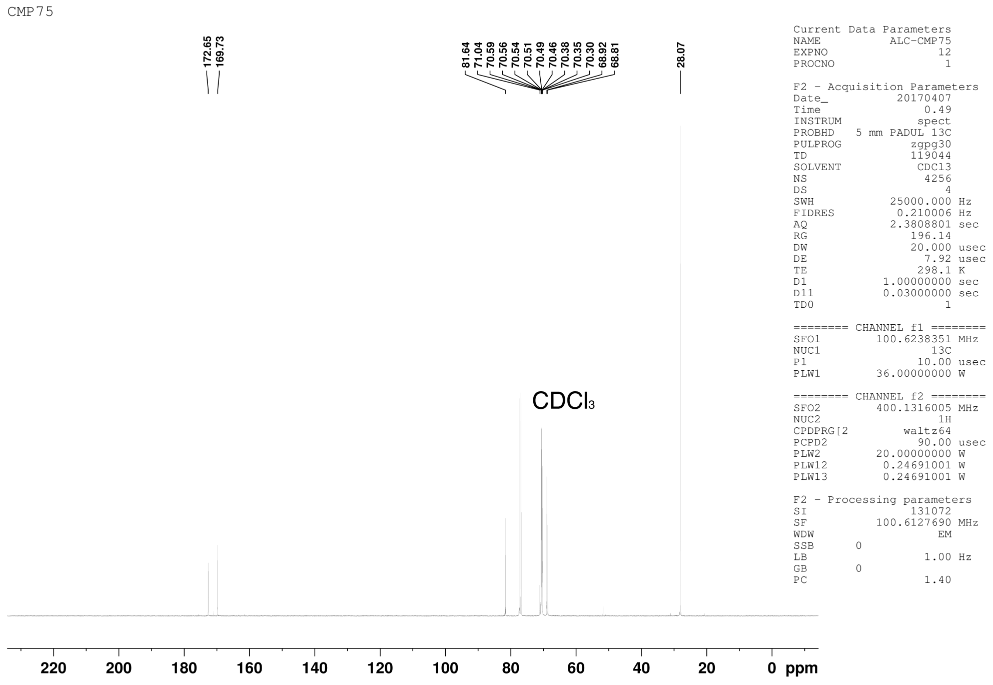

13, ^{1}H-NMR and ^{13}C-NMR in $CDCl_3$

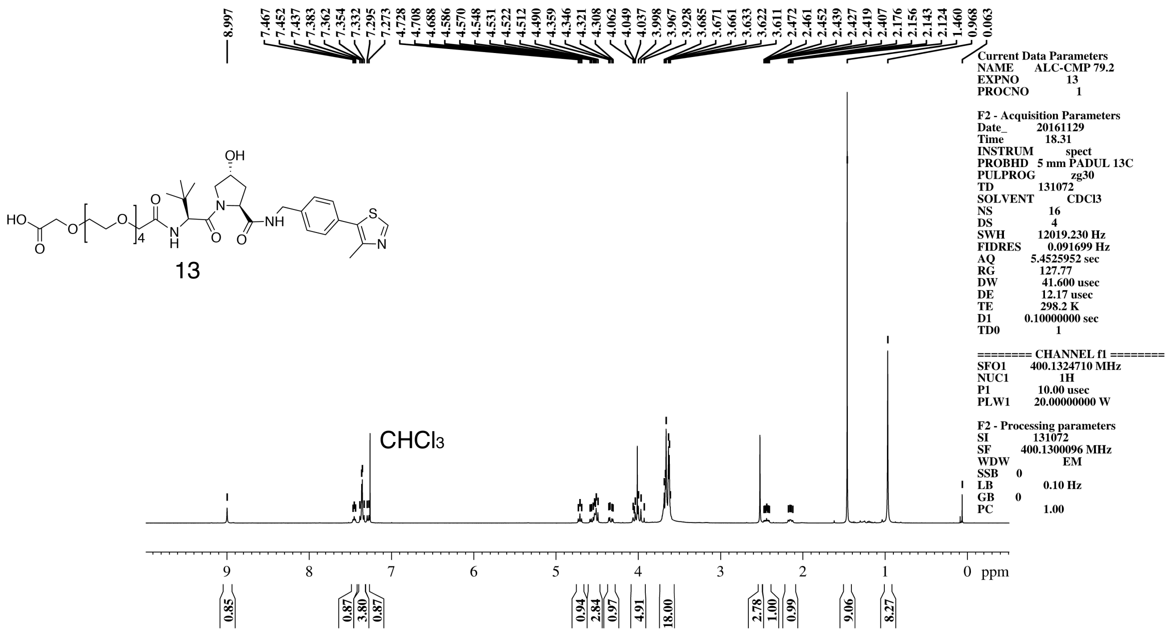

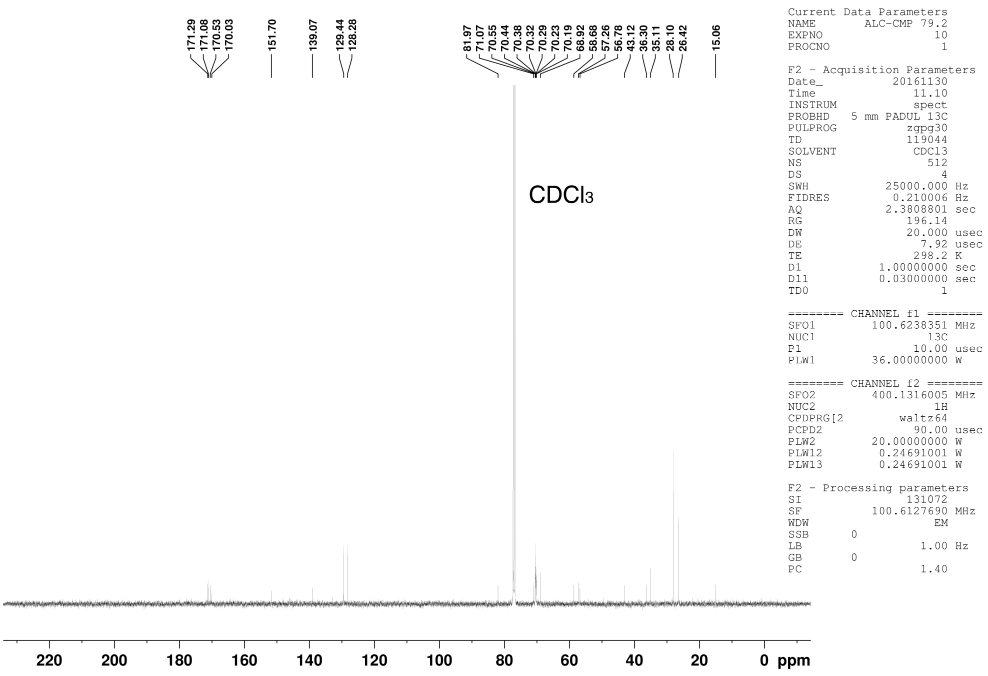

17, ^{1}H-NMR and ^{13}C-NMR in $CDCl_3$

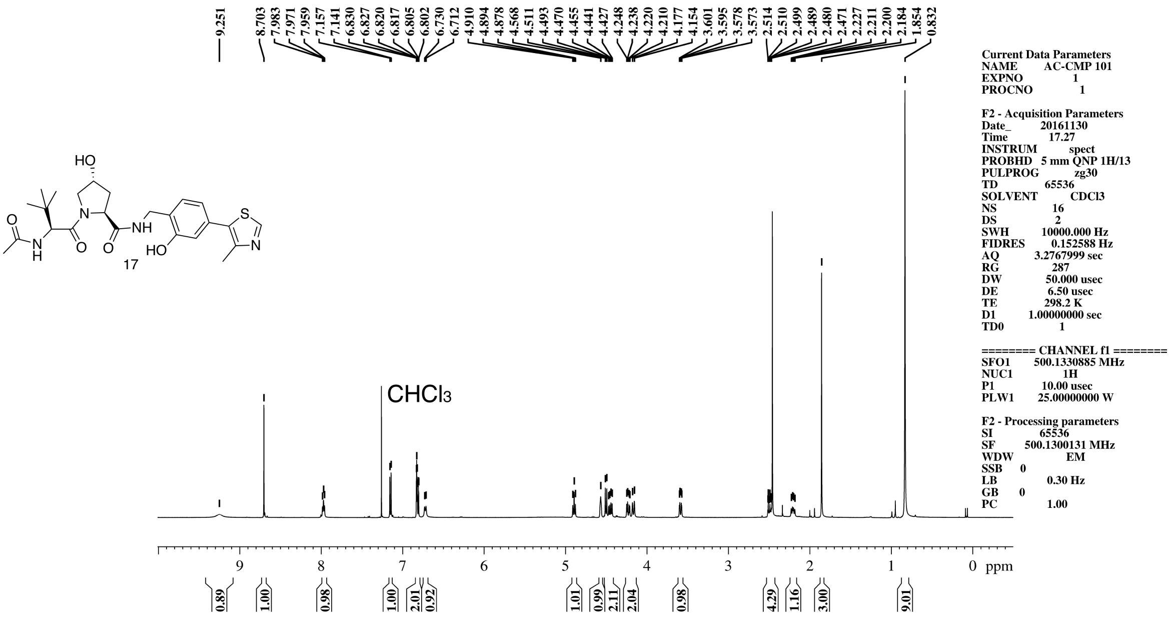

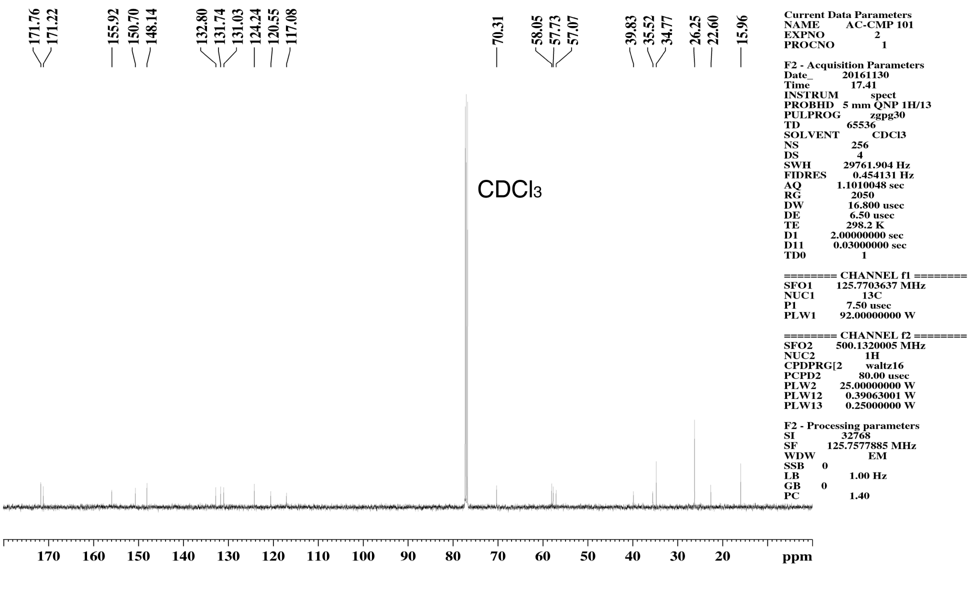

18, ^{1}H-NMR and ^{13}C-NMR in $CDCl_3$

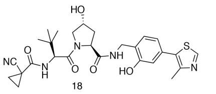

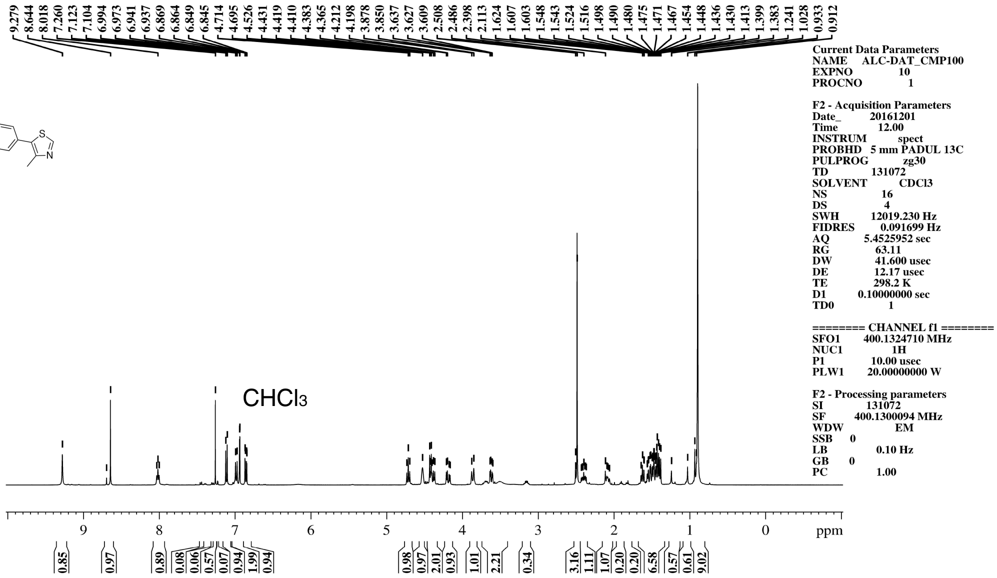

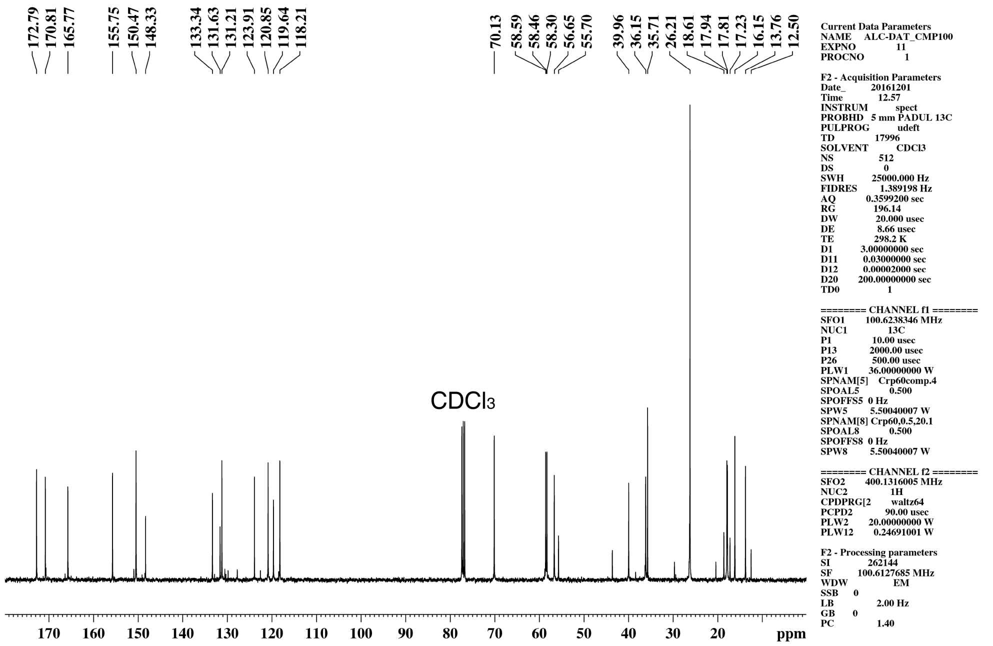

CMP106, ^{1}H-NMR and ^{13}C-NMR in $CDCl_3$

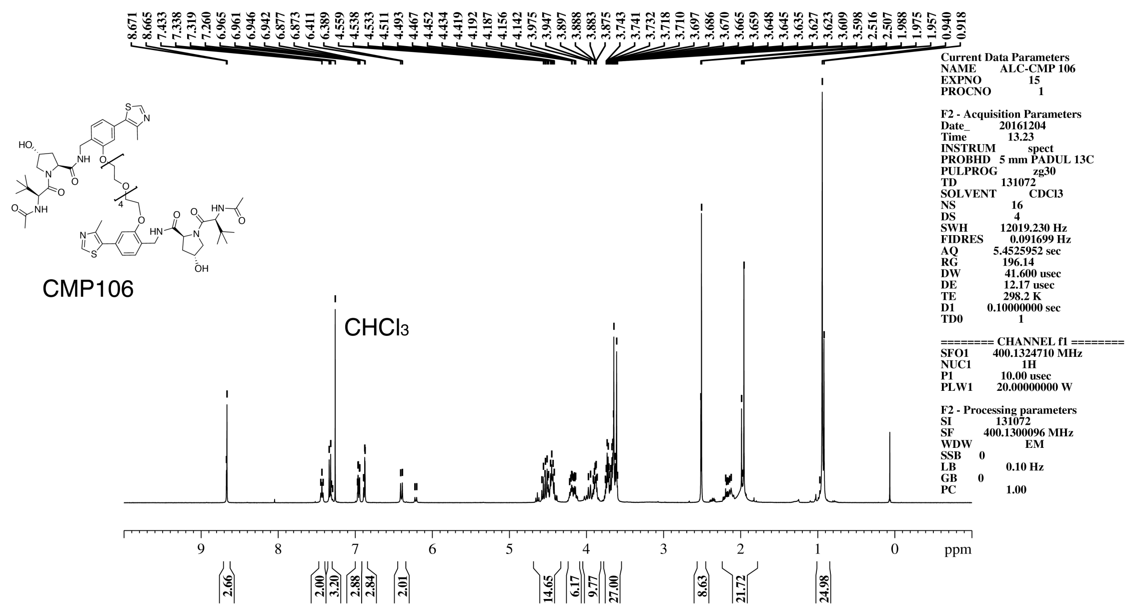

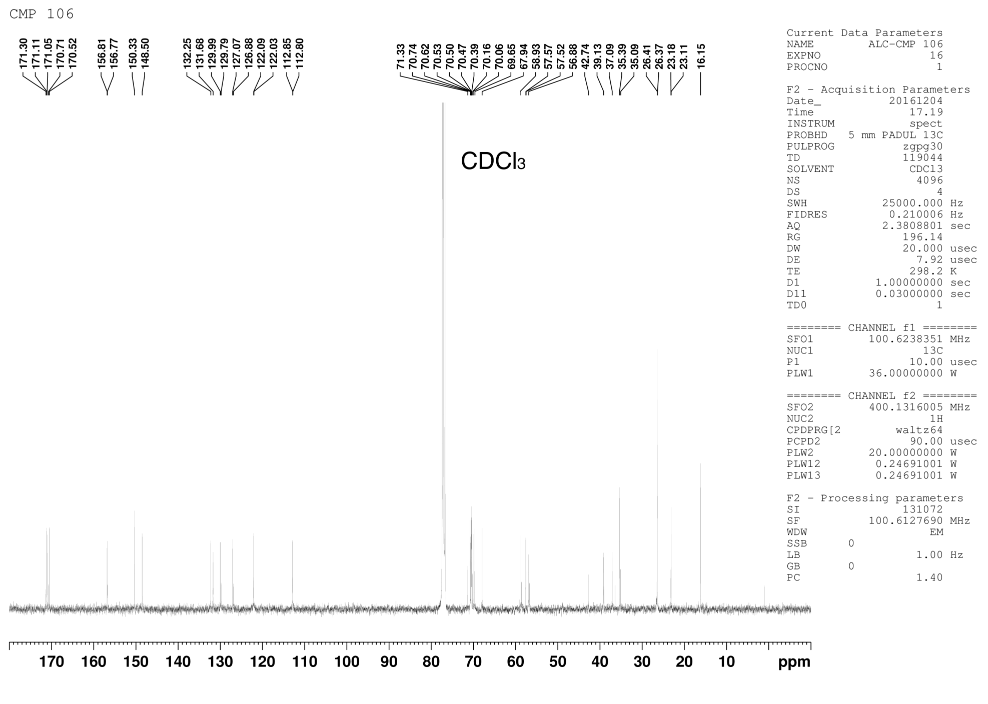

CMP108, ^{1}H-NMR and ^{13}C-NMR in $CDCl_3$

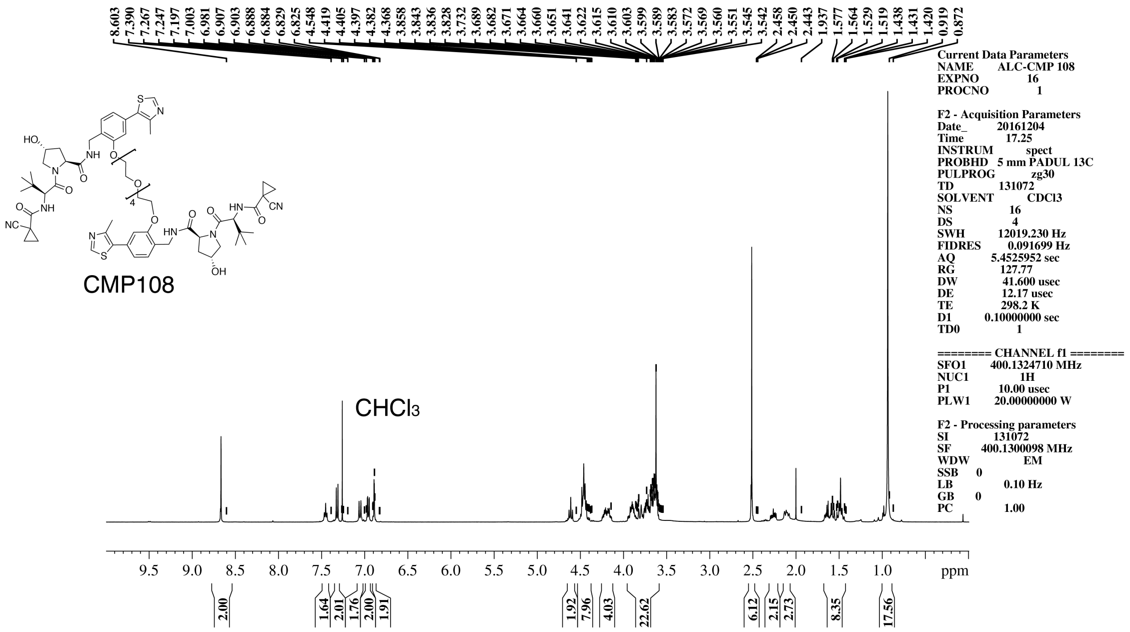

CMP108

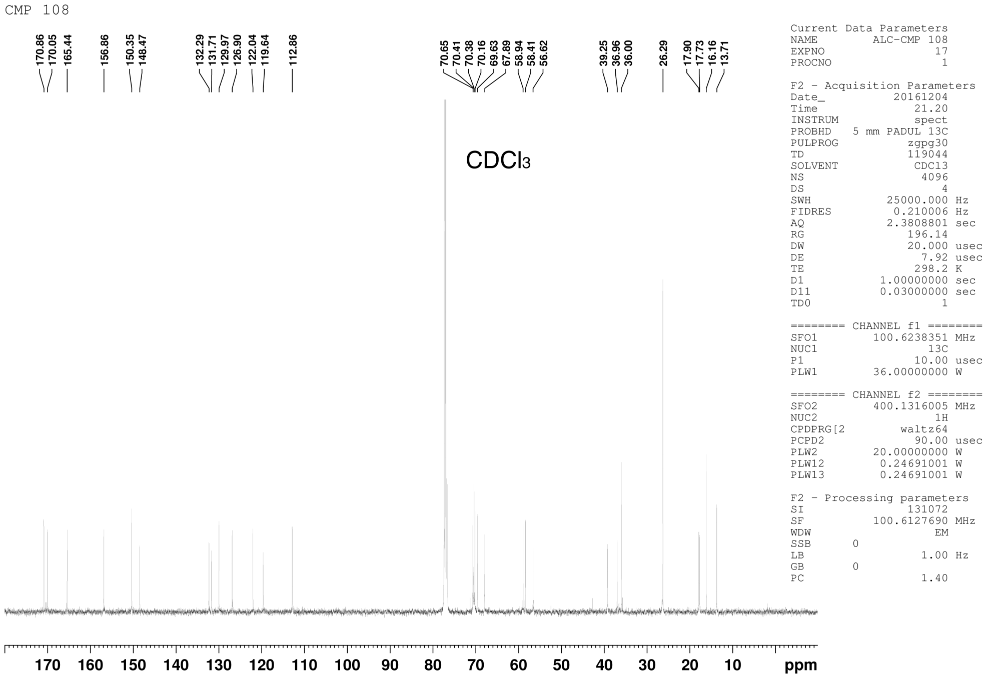

21, ^{1}H-NMR and ^{13}C-NMR in $CDCl_3$

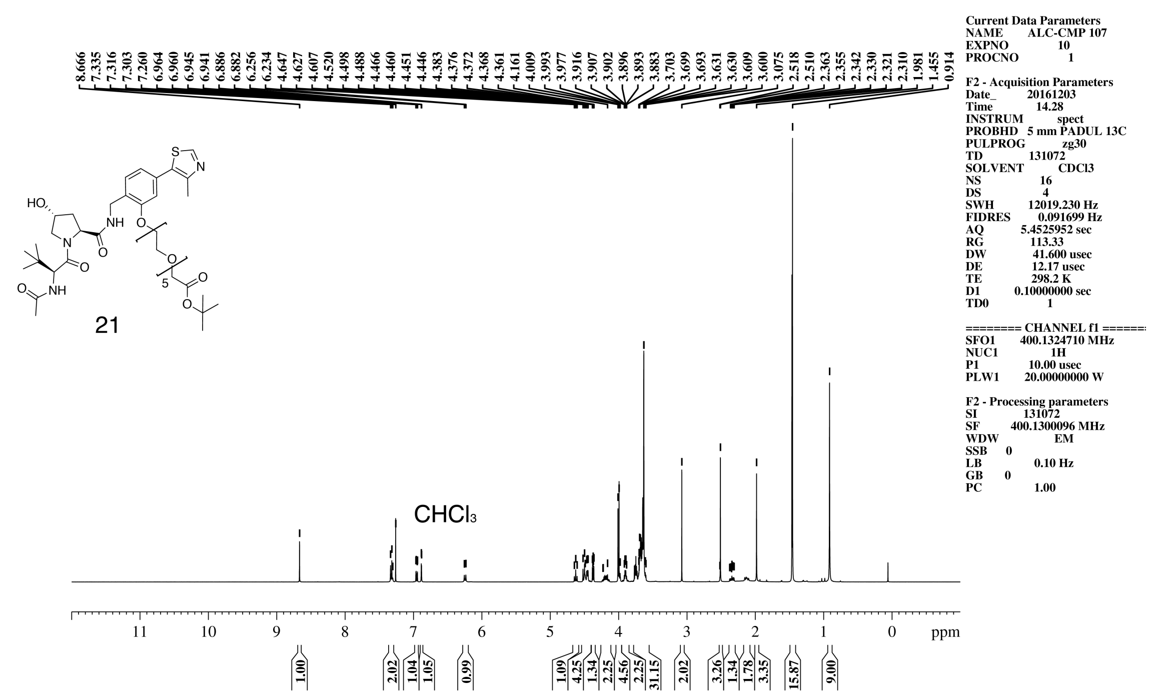

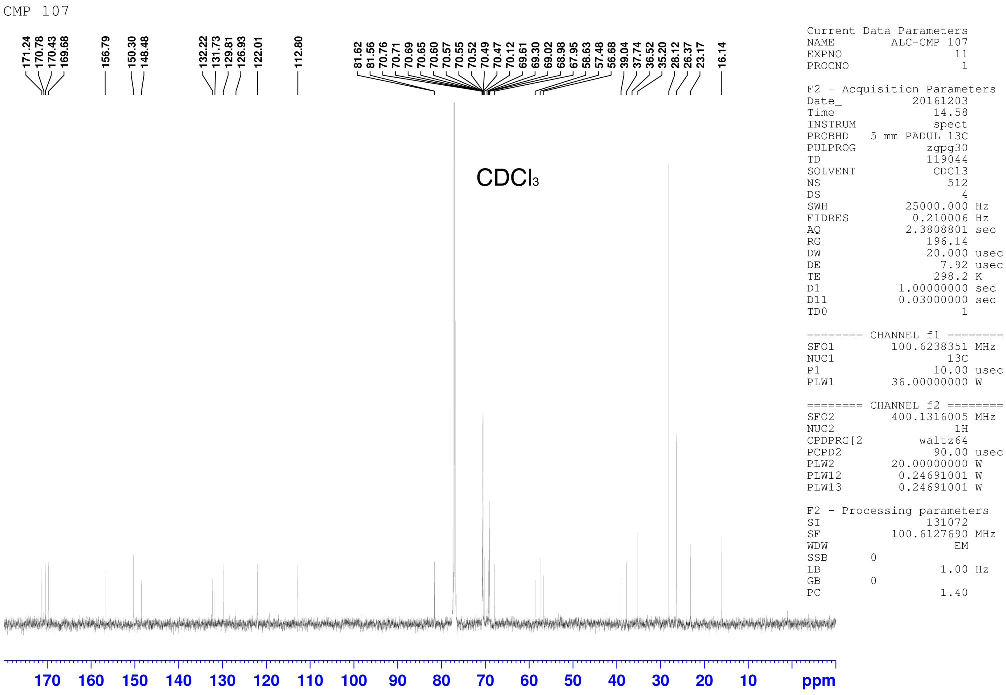

22, ^{1}H-NMR and ^{13}C-NMR in $CDCl_3$

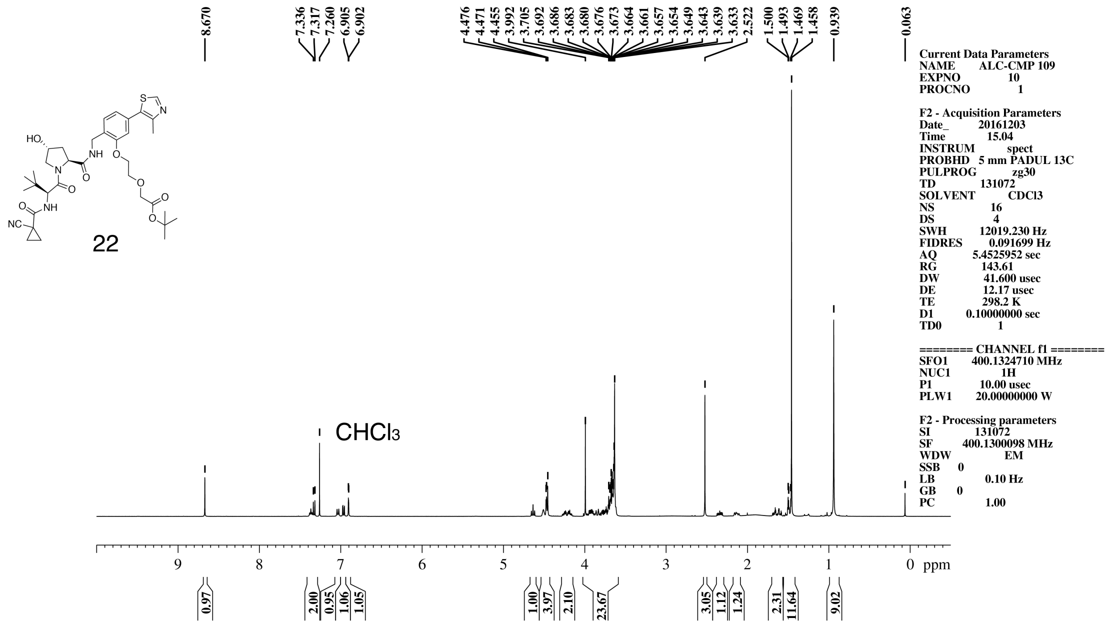

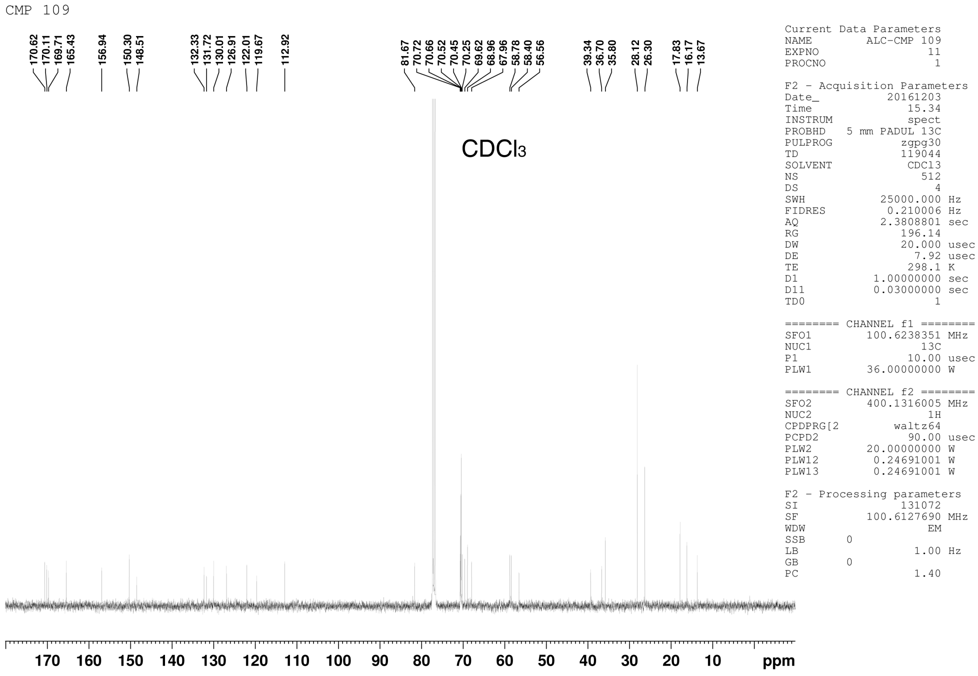

CMP112, ^{1}H-NMR and ^{13}C-NMR in $CDCl_3$

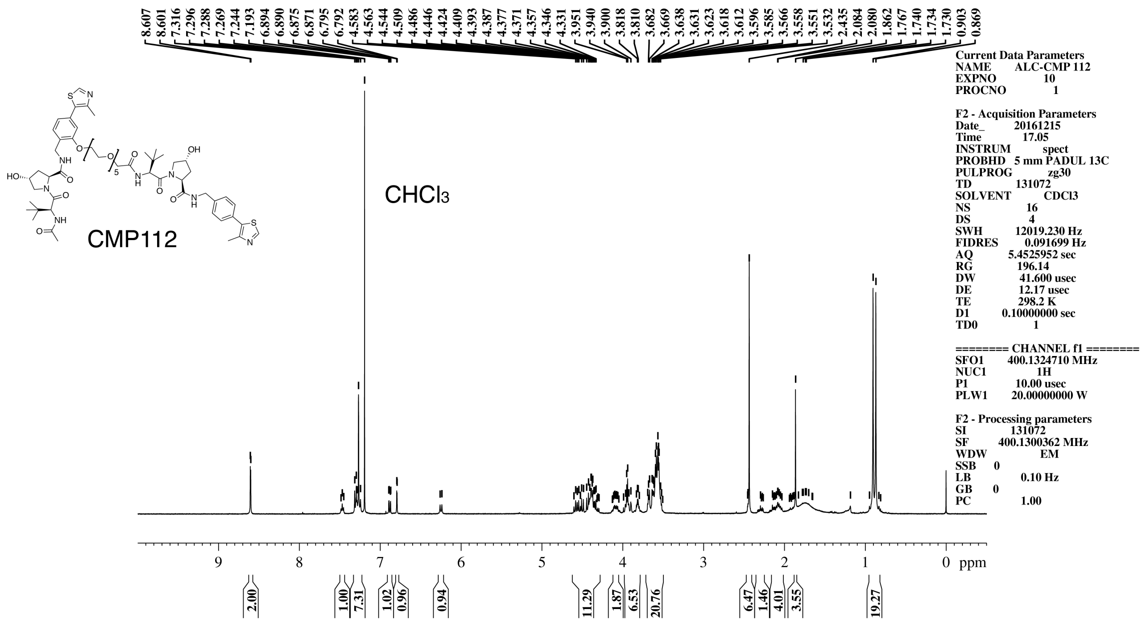

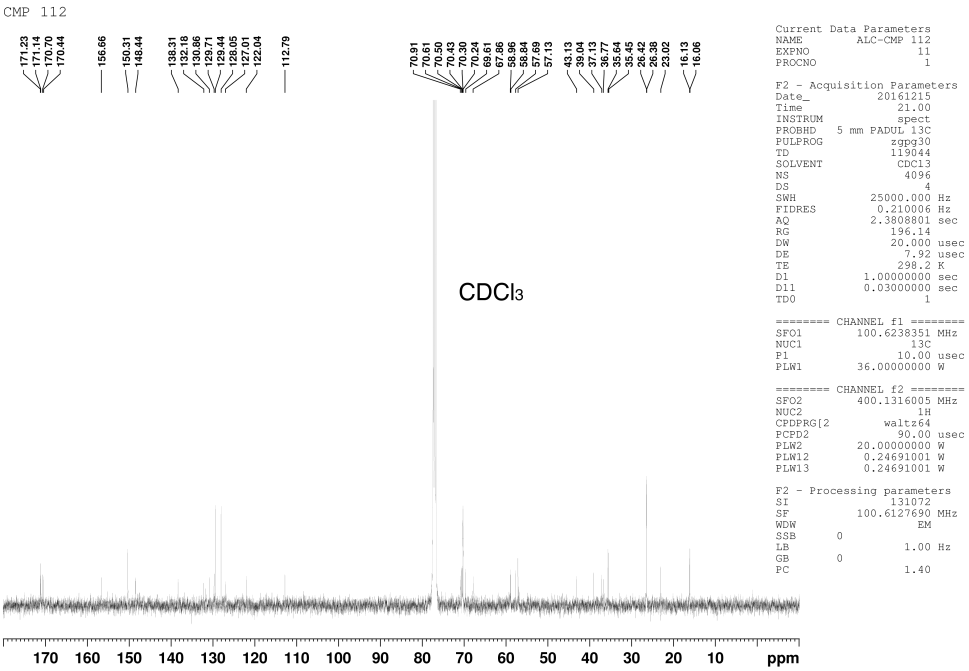

CMP113, ^{1}H-NMR and ^{13}C-NMR in $CDCl_3$

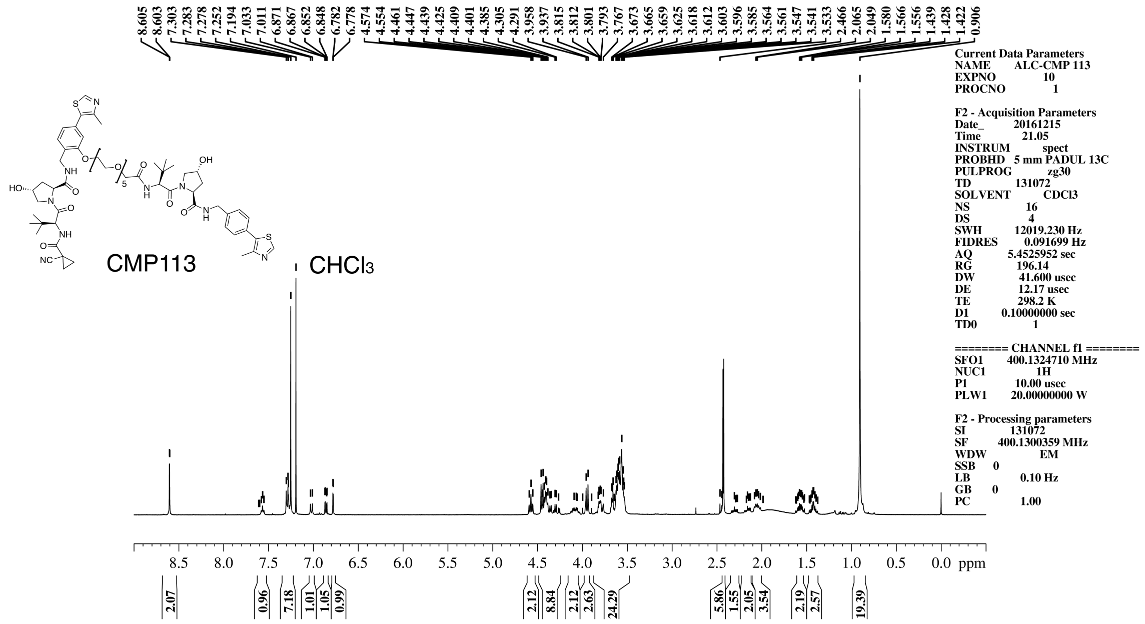

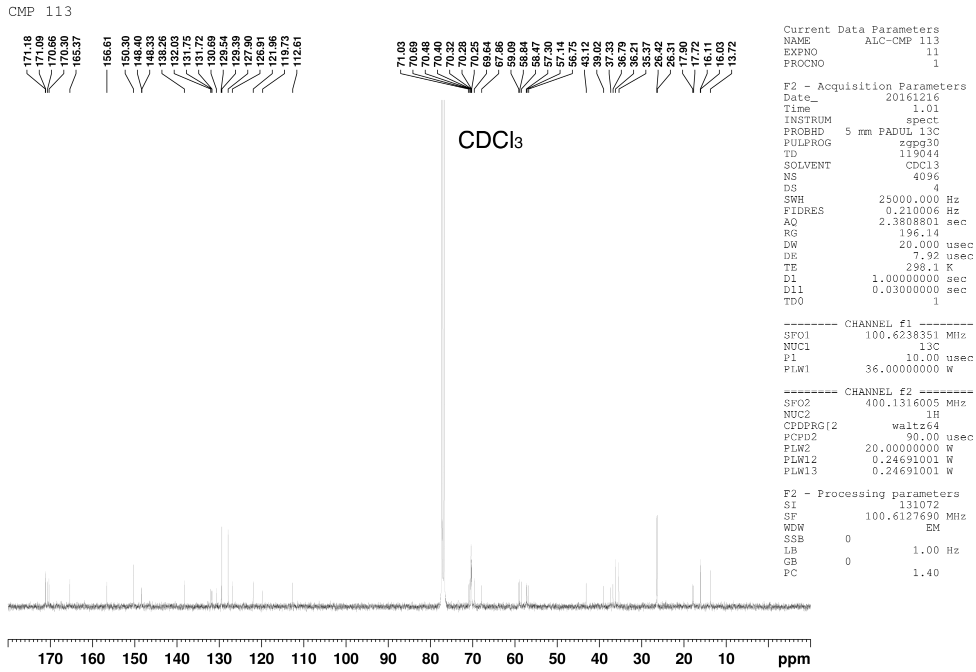

Supplementary References

1. Buckley, D. J. et al. HaloPROTACS: Use of Small Molecule PROTACs to Induce Degradation of HaloTag Fusion Proteins. *ACS Chem. Biol.* **10 (8)**, 1831-1837 (2015).

2. Kimura, Y., Miyabara, Y., Terashima, T. & Sawamoto, M. Polyacrylamide pseudo crown ethers via hydrogen bond-assisted cyclopolymerization. *J. Polym. Sci. Part A: Polym. Chem.* **54,** 3294–3302 (2016).

3. Arnold, L.D. et al. Bivalent bromodomain ligands, and methods of using same. WO 2015/081284, Intl. Publ date: 4 June 2015.
