# Supplementary material for: Homo-PROTACs: bivalent small-molecule dimerizers of the VHL E3 ubiquitin ligase to induce self-degradation
Source: Nat Commun. 2017 Oct 10;8:830. doi: 10.1038/s41467-017-00954-1 (PMC5635026; doi:10.1038/s41467-017-00954-1)
Supplement: Supplementary file 3 — Description of Additional Supplementary Files [file 41467_2017_954_MOESM3_ESM.pdf]

Description of Additional Supplementary Files

File Name: Supplementary Data 1
Description: Proteomic analysis of relative protein abundance in HeLa cells. Results for CRL / VHL related subunits (highlighted in yellow) are graphically represented in Supplementary Figure 6. Workbook "Output" is the output of the MaxQuant search as obtained. Workbook "ALL_filtered" lists quantified proteins filtered based on the criteria as outlined in the Online Methods.
